# A balancing act: heterochromatin protein 1a and the Polycomb group coordinate their levels to silence chromatin in *Drosophila*

**DOI:** 10.1186/s13072-015-0010-z

**Published:** 2015-04-30

**Authors:** Janel R Cabrera, Ursula Olcese, Jamila I Horabin

**Affiliations:** Department of Biomedical Sciences, College of Medicine, Florida State University, Rm 3300-G, 1115 W, Call St., Tallahassee, FL 32306 USA; Current Address: Center for Life Sciences, Department of Medicine, Division of Cardiology, Beth Israel Deaconess Medical Center, Rm 917, 3 Blackfan Circle, Boston, MA 02215 USA

**Keywords:** Heterochromatin, Euchromatin, Histone methylation, Gene expression, Polycomb, Heterochromatin protein 1a

## Abstract

**Background:**

The small non-histone protein Heterochromatin protein 1a (HP1a) plays a vital role in packaging chromatin, most notably in forming constitutive heterochromatin at the centromeres and telomeres. A second major chromatin regulating system is that of the Polycomb/trithorax groups of genes which, respectively, maintain the repressed/activated state of euchromatin. Recent analyses suggest they affect the expression of a multitude of genes, beyond the homeotics whose alteration in expression lead to their initial discovery.

**Results:**

Our data suggest that early in *Drosophila* development, HP1a collaborates with the Polycomb/trithorax groups of proteins to regulate gene expression and that the two chromatin systems do not act separately as convention describes. HP1a affects the levels of both the Polycomb complexes and RNA polymerase II at promoters, as assayed by chromatin immunoprecipitation analysis. Deposition of both the repressive (H3K27me3) and activating (H3K4me3) marks promoted by the Polycomb/trithorax group genes at gene promoters is affected. Additionally, depending on which parent contributes the null mutation of the HP1a gene, the levels of the H3K27me3 and H3K9me3 silencing marks at both promoters and heterochromatin are different. Changes in levels of the H3K27me3 and H3K9me3 repressive marks show a mostly reciprocal nature. The time around the mid-blastula transition, when the zygotic genome begins to be actively transcribed, appears to be a transition/decision point for setting the levels.

**Conclusions:**

We find that HP1a, which is normally critical for the formation of constitutive heterochromatin, also affects the generation of the epigenetic marks of the Polycomb/trithorax groups of proteins, chromatin modifiers which are key to maintaining gene expression in euchromatin. At gene promoters, deposition of both the repressive H3K27me3 and activating H3K4me3 marks of histone modifications shows a dependence on HP1a. Around the mid-blastula transition, when the zygotic genome begins to be actively transcribed, a pivotal decision for the level of silencing appears to take place. This is also when the embryo organizes its genome into heterochromatin and euchromatin. A balance between the HP1a and Polycomb group silencing systems appears to be set for the chromatin types that each system will primarily regulate.

**Electronic supplementary material:**

The online version of this article (doi:10.1186/s13072-015-0010-z) contains supplementary material, which is available to authorized users.

## Background

Nucleosomes package DNA into the basic unit of eukaryotic chromatin. They are repositioned or evicted by ATP-dependent chromatin remodeling enzymes or altered by covalent modification of their histone tails to mark the chromatin for activation or repression. Against this backdrop of chromatin states, gene expression is regulated and development and differentiation proceed.

While nucleosomes define the basic unit, at the genome level chromatin is frequently viewed as being organized into two major forms: euchromatin which is generally associated with gene-rich regions and the chromatin is less condensed, whereas heterochromatin is highly condensed, late replicating, and mostly transcriptionally silent throughout the cell cycle. It is generally gene poor and is associated with telomeres, centromeres, and pericentric regions.

A key component and contributor to the constitutive heterochromatin at telomeres and centromeres is heterochromatin protein 1 (HP1). HP1 is a small non-histone protein with two conserved domains, an N-terminal chromodomain which binds chromatin by recognizing methylated lysine 9 on histone H3 (H3K9) and a chromoshadow domain which enables HP1 to dimerize and bind to a variety of proteins through a degenerate pentapeptide motif, PxVxL [1]. It is also through the chromoshadow domain that HP1 interacts with H3K9 histone methylases (in *Drosophila* primarily Su(var)3-9 and Setdb1) to promote H3K9 methylation and facilitate the deposition and spreading of the mark (reviewed in [2]). HP1 is phylogenetically conserved and found in almost all eukaryotes, frequently as at least 3 isoforms (HP1a, HP1b, and HP1c in *Drosophila*). It is well known for its role in gene silencing and modifies position effect variegation in a dose-sensitive manner (position effect variegation (PEV); the process of gene silencing through the spread of heterochromatin, usually detected in rearranged chromosomes or transgene insertions). More recent studies, however, suggest HP1 also affects the transcription of euchromatin genes and can act positively [3] (reviewed in [4]).

A second major chromatin regulating system is that of the Polycomb/trithorax groups (PcG/trxG) of genes. PcG/trxG genes were first identified in *Drosophila melanogaster* as necessary for maintaining either the repressed/activated state, respectively, of the homeotics, genes which give segments their identity so patterning the body axis. Since their initial characterization, further analyses suggest that their function and regulation is widely conserved across metazoans. Additionally, high-throughput genome-wide techniques suggest that besides the homeotics, several hundreds, perhaps thousands of genes may be regulated by the PcG/trxG genes. Many of their targets appear to be essential developmental regulators [5,6].

The primary PcG/trxG players are evolutionary conserved in all higher eukaryotes [7-10]. PcG genes maintain silencing primarily by tri-methylating histone H3 at lysine 27 (H3K27me3) through the activity of Enhancer of zeste (E(Z)). E(Z) is in Polycomb repressive complex 2 (PRC2), along with three additional proteins, Suppressor of zeste 12 (SU(Z)12), Extra sex combs or Extra sex combs-like (Esc/Escl in *Drosophila*; *Eed* in mammals), and p55 (CAF1), which modulate the activity of the E(Z) methylase [11-13] (reviewed in [14]). PC itself is in PC repressive complex 1 (PRC1), which recognizes and spreads H3K27me3 to maintain the repressive state. Working against the PcG is the trxG in *Drosophila* and the homologous mixed lineage leukemia group in vertebrates, which maintains the active state through tri-methylation of H3K4 (H3K4me3) at the promoters of active genes (reviewed in [7]). Implicit from the various targets is that the PcG/trxG genes primarily regulate the genes in euchromatin.

The HP1 and PcG/trxG pathways are thought to have diverged and acquired different functions in maintaining genome stability in multicellular organisms. We find, however, that the two chromatin systems do not act separately. In the early *Drosophila* embryo, HP1a and the PcG proteins work together to regulate silencing and transcription. This interaction appears to be more acute during the early stages as later in development (larvae), HP1a appears to play less of a role in maintaining the levels of the PcG/trxG marks. Our data additionally suggest that the early *Drosophila* embryo has promoters which are bivalent with repressive H3K27me3 (PcG) and activating H3K4me3 (trxG) marks, both of which are dependent on HP1a. This dependence is also less clear cut in later stages. We speculate this effect of HP1a on the PcG and H3K27me3 levels may also occur in mammalian embryonic stem cells (ESC). We also find that as the early embryo organizes its genome into heterochromatin and euchromatin, the HP1/H3K9me and PcG/H3K27me3 systems appear to work together to both sense and partition the genome, sorting the chromatin type each will regulate. In the event of reduced maternal HP1a levels, the PcG system serves as a back-up resulting in an increase in H3K27me3 levels at heterochromatin, presumably to decrease the transcription of repetitive sequences which are derepressed by the reduction in HP1a. When H3K27me3 levels are low, there generally appears to be an increase in H3K9me3 levels, suggesting the two systems compensate for each other to achieve silencing. Collectively, these observations suggest the two chromatin systems work at multiple steps/stages to organize the genome.

## Results

In studying *Drosophila* sex determination, we found that HP1a plays both a repressive and activating role on the master switch gene, *Sex-lethal* (*Sxl*; [15]). The positive role suggested HP1a facilitates transcription initiation, as the levels of RNA polymerase II (RNAPII) at the *Sxl* establishment promoter, Sxl_Pe_, were adversely affected by reducing HP1a. We therefore tested whether HP1a plays a role in regulating another key developmental process and examined the homeotic gene *Ultrabithorax* (*Ubx*).

### Interaction of HP1a with PcG/trxG proteins at a homeotic gene

*Ubx* suppresses wing development to form halteres on the third thoracic segment of the fly. It is haploinsufficient and loss of one gene copy increases the haltere size, providing a sensitive readout of *Ubx* levels. Introducing a copy of the *Su(var)2-5*^*05*^ null mutation (HP1a gene name) in heterozygotes of the *Ubx*^*130*^ loss of function allele worsened the phenotype making the haltere larger (Additional file 1: Figure S1). This indicates a decrease in *Ubx* levels and that HP1a has a positive effect on *Ubx* expression, similar to what we observed at *Sxl*. It also demonstrates that a canonical PcG/trxG target is affected by HP1a. To confirm that the interaction was not from a background effect, a second allele (*Su(var)2-5*^*02*^) was examined and a similar though slightly weaker effect was observed (not shown). Our previous analyses with additional *Su(var)2-5* alleles (04 and 02 alleles) at *Sxl* also demonstrated *Su(var)2-5*^*02*^ had a weaker effect.

To further corroborate this interaction between the two different chromatin silencing systems, we decreased the dose of other PcG/trxG genes simultaneously with *Su(var)2-5*^*05*^and *Ubx*^*130*^ (Additional file 1: Figure S1). Introduction of *absent small and homeotic disks 1* (*ash1*), a member of the trxG of chromatin modifiers, improved the haltere phenotype suggesting an antagonistic relationship. This was somewhat surprising, as *ash1* normally promotes *Ubx* expression so its reduction should worsen the *Ubx*^*130*^ phenotype, which in the absence of the *Su(var)2-5*^*05*^ mutation was the case (Additional file 1: Figure S1). *E(z)*^*731*^ improved the haltere phenotype slightly, consistent with its repressive role. By contrast, *Su(z)12*^*4*^, slightly enhanced the effect of *Su(var)2-5*^*05*^ suggesting the two mutations promote each other’s effect, indicating a mild positive role of *Su(z)12* on *Ubx*. The canonical role of SU(Z)12 in PRC2 is to promote the methylase activity of E(Z) so it should behave like E(Z), which was the case in the absence of the *Su(var)2-5*^*05*^ mutation. This unexpected positive effect of *Su(z)12* suggested by the *Su(var)2-5*^*05*^*Ubx*^*130*^ phenotype could be from an additional role with HP1a as, unlike E(Z), SU(Z)12 also regulates heterochromatin and affects PEV [16]. Taken together, these results demonstrate that the various PcG/trxG proteins and HP1a interact to affect *Ubx* expression. The interactions are complex, uncovering unexpected changes in expression levels.

### HP1a has dual effects on the transcription of euchromatin genes

**Figure 1:**
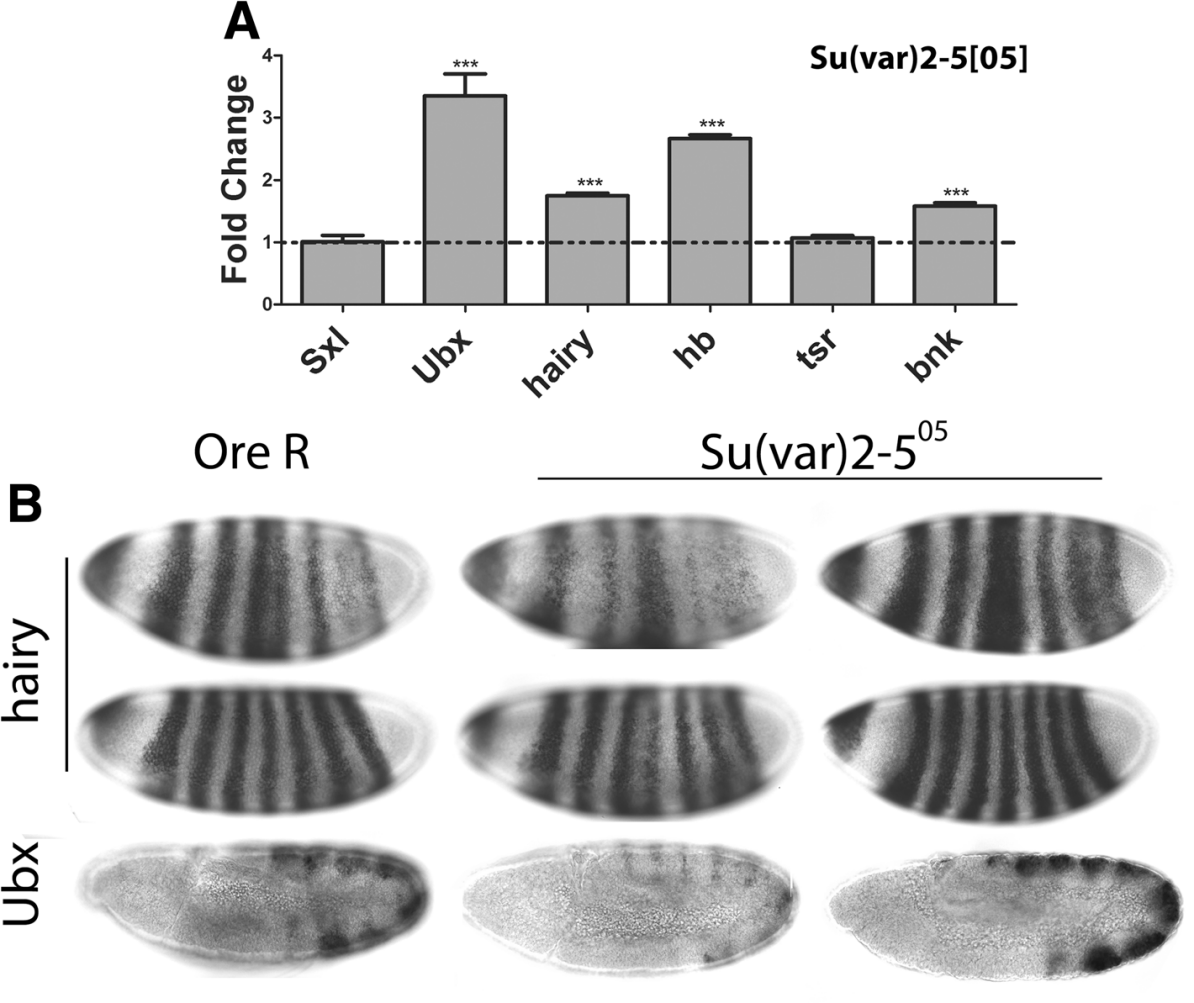
HP1a has dual roles in regulating transcription of *Drosophila* genes. **(A)** mRNA levels for six genes in 2–3 h embryos from *Su(var)2-5* ^*05*^ */CyO* parents relative to *Ore R* (set to one, dotted line). Asterisks show changes which are significant relative to *Ore R* (***P value <0.0005); for *tsr* and *Sxl* the difference is not significant. **(B)** *In situ* hybridization for *h* and *Ubx* in embryos from *Ore R* or *Su(var)2-5* ^*05*^/*CyO* parents. Top two rows show two different stages as the seven stripes of *h* resolve; in both cases, embryos with a decrease or increase in signal relative to wild-type are seen in the *Su(var)2-5* ^*05*^ class. Third row shows similar effects on *Ubx* which had a larger disparity. Weaker, stronger, and normal expression embryos 20:41:22 for *h*; 10:16:8 for *Ubx*; proportions of approximately 1:2:1. Sxl, Sex-lethal; Ubx, Ultrabithorax; hb, hunchback; tsr, twinstar; bnk, bottleneck.

The genetic interactions described above prompted us to examine whether HP1a regulates the transcription of other genes. The mRNA levels of six genes (*Sxl*, *Ubx*, *hairy* (*h*), *bottleneck* (*bnk*), *hunchback* (*hb*) and *twinstar* (*tsr*)) were quantified in 2–3 h embryos from wild-type and *Su(var)2-5*^*05*^ heterozygous parents (Figure 1A). Relative to wild-type, their levels were 1.0, 3.3, 1.7, 1.6, 2.7, and 1.1, respectively, indicating a derepression (or no effect in the case of *Sxl*), which is in keeping with the canonical role of HP1a as a repressor. However, prior *in situ* hybridization data had demonstrated that HP1a has a positive role on *Sxl*, with is reduction severely inhibiting *Sxl* expression [15]. This suggested that the value of 1 relative to wild-type for *Sxl* mRNA must arise from some of the embryos having higher than normal expression, to compensate for the embryos with poor to low expression. Indeed, the prior *in situ* hybridizations had suggested slightly higher than normal expression in close to 50% of the older embryos, primarily in cycle 14 but not cycle 13 embryos, suggesting a zygotic effect in the embryos which have a wild-type chromosome.

As *in situ* hybridization allows observation of individual embryos which the analysis of bulk mRNA does not, we analyzed three additional genes (*Ubx*, *h*, *bnk*) (Figure 1B). Unlike wild-type embryos which gave fairly uniform signal levels for a given stage, the embryos from the heterozygous *Su(var)2-5*^*05*^ parents were not equally affected. Three classes could be observed: one in which the expression is weaker, a second with near normal levels, and a third in which the expression is slightly stronger (Figure 1B). We suspect the weak class are the homozygous *Su(var)2-5*^*05*^ embryos, the stronger class are *Su(var)2-5*^*05*^ heterozygotes, and the normal looking embryos are homozygous for the balancer with two normal copies of *Su(var)2-5* (proportions of 1:2:1 support this claim; *n* = 83 for *h*; *n* = 34 for *Ubx*). Quantifying the embryos from the *bnk in situ* hybridizations was challenging as its expression changes within the cell cycle; however, comparisons of embryos at the same stage in the cell cycle showed some embryos with either weaker or stronger than wild-type signal (Additional file 1: Figure S2) suggesting effects similar to those seen at *Ubx*, *h*, and *Sxl*. Together, these data suggest that (i) HP1a heterozygotes show an increase in expression, presumably from a decrease in repression while (ii) severely reducing/eliminating HP1a adversely affects expression. This dual effect emphasizes that HP1a has more than one role in the transcriptional regulation of euchromatin genes and is consistent with our previous observations of an activating and repressing role at *Sxl* [15].

### Transcriptional repression by HP1a through H3K27me3

**Figure 2:**
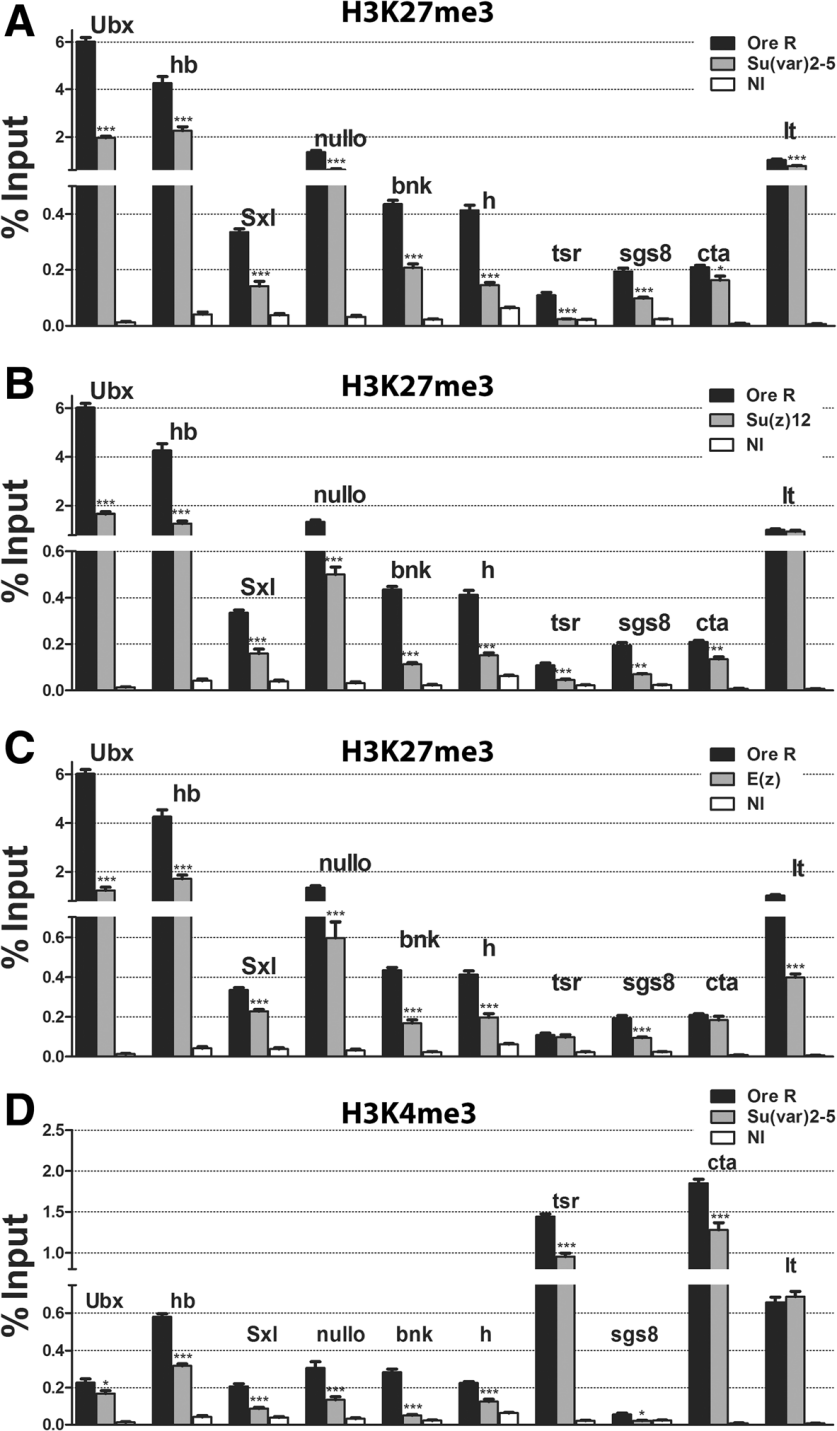
HP1a affects levels of H3K27me3 and H3K4me3. **(A-C)** H3K27me3 levels in 1–3 h embryos from **(A)** *Su(var)2-5* ^*05*^ */CyO*, **(B)** *E(z)* ^*731*^ */TM6*, and **(C)** *Su(z)12* ^*4*^ */TM6* parents at promoters of *Ubx*, *hb*, *Sxl*, *nullo*, *bnk*, *h, tsr*, *sgs8*, *cta*, and *lt*. All NI data is significantly below the lowest ChIP signal. **(D)** H3K4me3 levels 1–3 h embryos from *Su(var)2-5* ^*05*^ */CyO* parents at same promoters. Asterisks show significant changes relative to *Ore R* (*P value <0.05, **P value <0.005, ***P value <0.0005). Error bars represent ± SEM. H3K27me3, trimethylated histone H3 at lysine 27; NI, non-immune; Sxl, Sex-lethal; Ubx, Ultrabithorax; hb, hunchback; tsr, twinstar; bnk, bottleneck; h, hairy; lt, light; cta, concertina.

Given that HP1a is necessary for proper *Ubx* expression and it appears to repress several functionally different genes, we wondered if the PcG/trxG of chromatin modifiers were working together with HP1a. To answer this question, chromatin immunoprecipitation using antibodies specific for H3K27me3, followed by quantitative PCR (ChIP-qPCR), was performed on 1–3 h embryos from wild-type and *Su(var)2-5*^*05*^ heterozygous parents (Figure 2A). The promoters of ten genes with varying functions were scored; they include a highly expressed housekeeping gene (*tsr*), segmental patterning (*Ubx*, *hb*, *h*), sex determination, cellular membrane organization genes (*bnk*, *nullo*), and genes residing in facultative heterochromatin (*concertina* (*cta*) and *light* (*lt*)). *sgs8*, the salivary gland specific gene, represents non-expressed genes. All showed a significant reduction in their H3K27me3 levels in the *Su(var)2-5*^*05*^ background (Figure 2A). Compellingly, the decrease by *Su(var)2-5*^*05*^ is very comparable to reducing the H3K27 methylase itself, E(Z), or its binding partner, SU(Z)12 (Figure 2B,C). We note that the H3K27me3 signal strength in wild-type embryos is similar to other studies using *Drosophila* S2 cells or slightly older embryos, and for genes in common, the signal is almost identical [17-20]. Second, the ChIPs show each gene has a different signal strength, underscoring the fact that each gene is unique for the mark. Third, while some of the genes have a relatively low signal, it is further reduced (but is still above background) by *Su(var)2-5*^*05*^, indicating it is real.

As the *Su(var)2-5*^*05*^ embryos show a decrease in H3K27me3, we tested whether the opposite mark promoted by the trxG, H3K4me3, was also affected. ChIP-qPCR was performed on 1–3 h embryos from wild-type and *Su(var)2-5*^*05*^ heterozygous parents. Of the promoters tested, loss of HP1a significantly reduced H3K4me3 levels at nine genes (Figure 2D); *lt* was unchanged although *cta*, which is also in heterochromatin, did show a drop. ChIP-qPCR in embryos from heterozygous *Su(z)12*^*4*^ or *E(z)*^*731*^ parents did not show this consistent decrease in H3K4me3 (Additional file 1: Figure S3), suggesting that HP1a is unique in this respect.

That these effects are from the reduction of HP1a and not from the H3 methylases or a background effect is suggested by several controls (Additional file 1: Figure S4). First, Western blot analysis on 1–2 h embryos shows that the levels of maternal HP1a protein are less in the *Su(var)2-5*^*05*^embryos than wild-type (Additional file 1: Figure S4A). Second, qRT-PCR analysis to measure the levels of *E(z)* mRNA (and the two major H3K9 methylases) shows that there is more message in the *Su(var)2-5*^*05*^embryos (Additional file 1: Figure S4B). Western blots of E(Z) protein show its levels are comparable to wild-type, however (Additional file 1: Figure S4A). This suggests that it is not the methylase but another activity which is limiting the deposition of H3K27me3. Third, when HP1a levels are reduced using RNA interference, we also find a decrease in H3K27me3 (Additional file 1: Figure S4C). Finally, introducing a copy of an RFP-HP1a transgene into the *Su(var)2-5*^*05*^*/CyO* background improves or rescues the reduction in *both* H3K27me3 and H3K4me3 at almost all the promoters (Additional file 1: Figure S4D,E). The main exceptions were: for H3K27me3, *hb* and the heterochromatin genes (*lt* and *cta*); for H3K4me3, most indicated a rescue with some genes showing a slight overshoot, except for *lt* which decreased*.* The RFP-HP1 transgene was also effective at correcting the elevated levels of *Ubx*, *h*, and *bnk* mRNA in 2–3 h embryos (Additional file 1: Figure S4F). This suggests that the transcriptional effect is also due to a reduction in HP1a. (*hb* was an exception and actually increased. Consistent with this elevated transcription, *hb* had its H3K27me3 levels unchanged while its H3K4me3 levels were more than fully rescued by the RFP-HP1a transgene (Additional file 1: Figure S4D).)

The RFP-HP1a transgene uses *Su(var)2-5*^*05*^genomic sequences to drive expression and is maternally expressed (Additional file 1: Figure S4A). It is unable to fully rescue the *Su(var)2-5*^*05*^mutation to viability however, perhaps because the RFP, which is as large as HP1a itself, blocks the amino terminus of HP1a which may alter its normal transcriptional function. Nevertheless, the significant improvements we observe in the different effects of HP1a, including the repetitive elements with low H3K27me3 levels in the 1–3 h window (1360 and HeT-A; Additional file 1: Figure S4D,E), support the idea that the changes in the *Su(var)2-5*^*05*^embryos are from a reduction in HP1a and not from changes in the histone methylases or some unknown background effect.

### HP1a more consistently affects gene promoters

**Figure 3:**
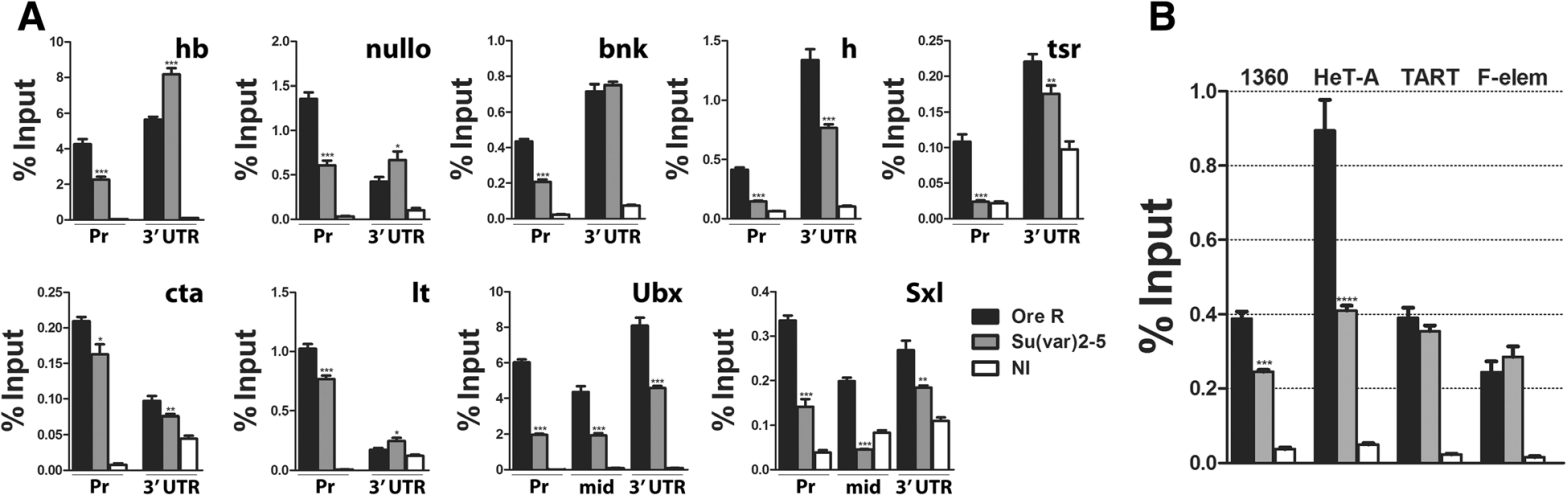
Reduction in HP1a consistently reduces H3K27me3 levels at gene promoters. **(A)** Levels of H3K27me3 in 1–3 h embryos at different gene regions in addition to their promoters. Heterochromatin is represented in **(B)** by the 1360 repetitive element, TART and HeT-A telomeric elements, and the F-element found in pericentric heterochromatin as well as other heterochromatic regions across the genome. Most of the genes (five of the nine) show a decrease also at their 3′ ends and for *Sxl* and *Ubx*, 1 kb downstream of the promoter (labeled mid). Three of the nine (*hb*, *nullo*, and *lt*) show an increase while *bnk* did not show a change. Two of the repetitive regions are unchanged (TART, F-element) while the remaining two show a decrease (1360, HeT-A). Asterisks show changes which are significant different from wild-type (*P value <0.05, **P value <0.005, ***P value <0.0005). Error bars represent ± SEM. NI, non-immune; Sxl, Sex-lethal; Ubx, Ultrabithorax; hb, hunchback; tsr, twinstar; bnk, bottleneck; h, hairy; lt, light; cta, concertina.

The combined data above suggest that HP1a is upstream of the PcG/trxG proteins and its reduction affects both the repressive H3K27me3 and active H3K4me3 marks. To test if the effect is global, we examined the H3K27me3 levels in early embryos by Western blots. Unexpectedly, the levels did not appear significantly different in the *Su(var)2-5*^*05*^ embryos (Additional file 1: Figure S5), suggesting that while the promoter regions show a consistent reduction, the effect of HP1a may be local or that other regions in the genome have higher levels of H3K27me3 to offset the drop. To differentiate between these scenarios, the 3′ UTR regions of nine of the ten genes were scored. For *Ubx* and *Sxl*, the two largest genes, a region 1 kb downstream from the promoter was also scored. Different heterochromatic sequences were also examined - the 1360 repetitive element, TART and HeT-A telomeric elements, and F-element which is abundant in pericentric heterochromatin as well as other heterochromatic regions across the genome. As seen in Figure 3A, the decrease in H3K27me3 in most of the gene promoters (five of the nine) is also seen at the gene 3′ ends and for *Sxl* and *Ubx*, 1 kb downstream of the promoter. Three of the nine (*hb*, *nullo*, and *lt*) showed an increase, while *bnk* did not show a change, indicating that these gene regions are not equally affected by the reduction in HP1a. For the repeated elements, 1360 and Het-A were reduced, while TART and F-element did not show a change (Figure 3B). Taken together, these results suggest that reducing HP1a can affect the H3K27me3 levels at multiple regions in the genome, often showing a decrease, but increases are also seen. Promoters, however, appear to show a consistent decrease, and as these are critical to expression they were further analyzed.

**Figure 4:**
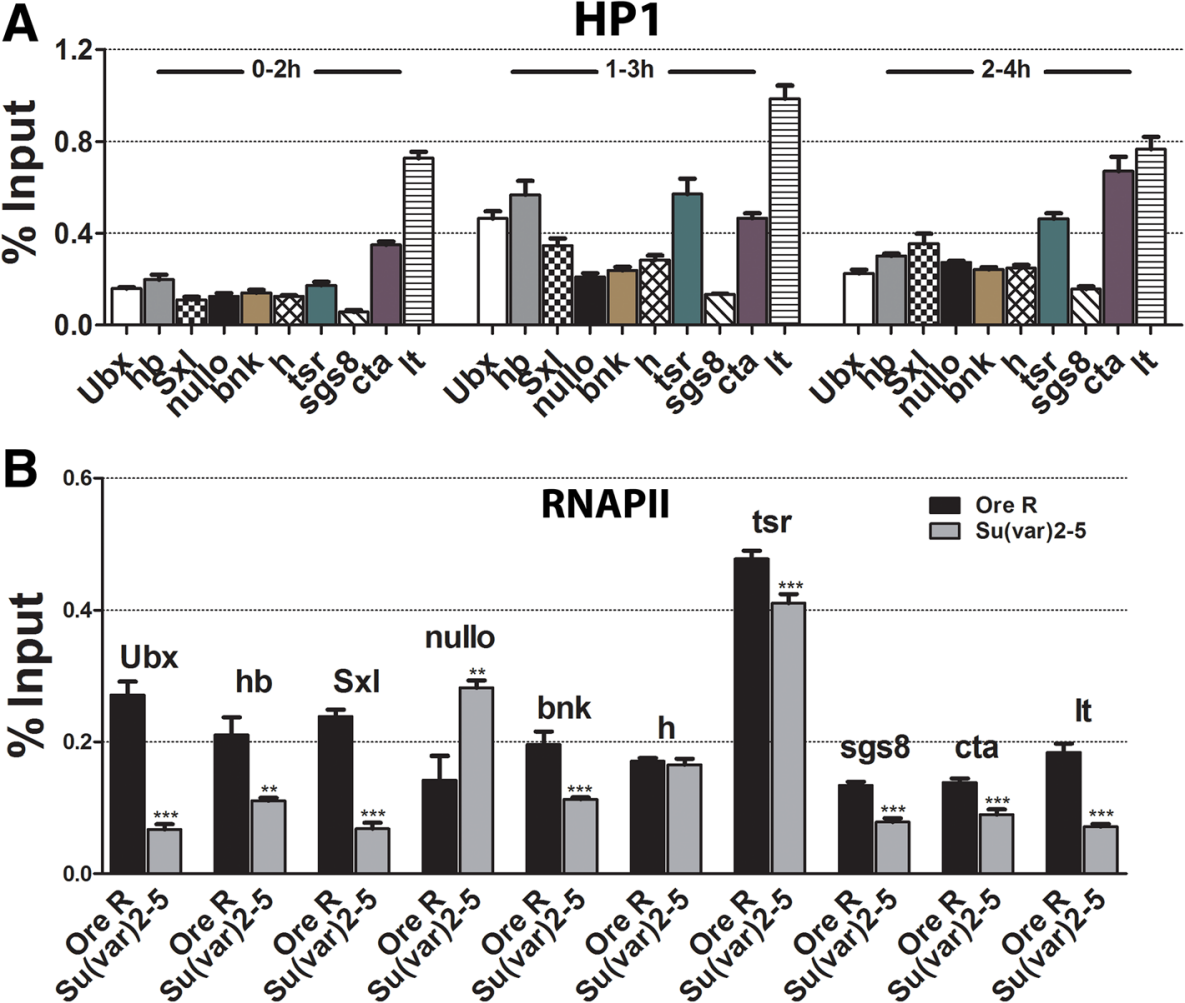
HP1a is at gene promoters and its reduction generally decreases the amounts of Ser-5P RNAPII at promoters. **(A)** HP1 ChIP data from 0–2, 1–3, and 2–4 h wild-type embryos at the promoters of *Ubx*, *hb*, *Sxl*, *nullo*, *bnk*, *h, tsr*, *sgs8*, *cta*, and *lt*. All show an increase after 0–2 h. **(B)** ChIP data for Ser-5P RNAPII from 1–3 h embryos from wild-type and *Su(var)2-5* ^*05*^ */CyO* parents. Eight of the ten promoters show a significant decrease. The *h* promoter was unchanged, while the *nullo* promoter showed an increase. Asterisks show changes which are significant relative to *Ore R* (**P value <0.005, ***P value <0.0005). Error bars represent ± SEM. HP1, heterochromatin protein 1; RNAPII, RNA polymerase II; Sxl, Sex-lethal; Ubx, Ultrabithorax; hb, hunchback; tsr, twinstar; bnk, bottleneck; h, hairy; lt, light; cta, concertina.

If HP1a modulates the levels of the PcG/trxG protein modifications at gene promoters, this raises the question of whether the protein is at the promoters. ChIP-qPCR on wild-type embryos using antibodies to HP1a shows the protein is indeed present at all the promoters we examined (Figure 4A). We speculate that this HP1a is what recruits the PcG/trxG chromatin modifying complexes. ChIP-qPCR for phospho Ser-5 at the RNAPII C-terminal domain (Ser-5P RNAPII) at the same promoters shows that the reduction of HP1a generally reduces the levels of this RNAPII isoform; eight of the ten promoters had a significant reduction with the exception of *h*, which was unchanged, and *nullo*, which was elevated (Figure 4B). Previously, we demonstrated that HP1a facilitates the recruitment, stability, or pausing of RNAPII at *Sxl*_*Pe*_; these results support and extend this prior observation [15]. Many of the promoters we analyzed are paused [21], so a reduction in HP1a may promote RNAPII release and increase transcription. We note that the specific change HP1a causes on RNAPII is outside the scope of this manuscript, beyond documenting its effects on the transcriptional machinery.

### Genes with low H3K27me3 levels are PcG/trxG targets

Genes with high levels of H3K27me3 are readily recognized as PRC2 targets, however depending on the threshold, classification of genes with lower levels as *bona fide* targets is debatable. *Ubx* and *hb* clearly have high levels of H3K27me3 in embryos (Figure 2), but the remaining genes, particularly *Sxl* or *cta*, have much lower levels. As *Sxl* is exquisitely dose-sensitive, we investigated whether its expression is affected by the PcG/trxG proteins to determine if it is a true PcG/trxG target.

Mutations in PcG/trxG proteins were first tested for genetic interactions with sex determination genes. Sex in *Drosophila* is determined by the number of X chromosomes, balanced against maternal factors and the sets of autosomes. Early in development (approximately 2–3 h of embryogenesis), this balance is deciphered by the *Sxl* establishment promoter, Sxl_Pe_, to result in its transcription in only females (XX animals; [22]). This activation of Sxl_Pe_ initiates a splicing feedback loop that maintains SXL expression and sexual identity through the rest of the life cycle [23,24] (reviewed in [25]).

*E(z)*, *Su(z)12*, and *ash1* mutants were tested against a sensitized background of reduced female promoting X-linked genes (*sisterless-a* (*sis-a*), *sisterless-b* (*sis-b*)). In this setup, an additional mutation can either worsen or improve female viability depending on whether the gene acts, respectively, positively or negatively in the process (female viability is affected as SXL turns off the dosage compensation system which hypertranscribes the X chromosomes by default). A reduction of *ash1* strongly compromised female viability suggesting it facilitates Sxl_Pe_ expression. This lethality was completely reversed by introducing a constitutive allele of *Sxl*, supporting the idea that *ash1* is needed for *Sxl* activation and not its maintenance (Additional file 1: Table S1 and Figure S7). Decreasing the levels of *E(z)* and *Su(z)12* improved female viability suggesting they act negatively (Additional file 1: Tables S1 and S2). This is consistent with the opposing activities of *ash1* and *Su(z)12*/*E(z)* and the idea that PcG/trxG proteins regulate the sex determination decision.

Performing additional genetic interaction tests with *Sxl* indicates the two chromatin modifying systems can strongly synergize. Mutation of *Su(var)3-9* alone weakly enhances female viability suggesting it acts negatively, which is in stark contrast to the poor viability of females with reduced HP1a. However, for all genes, the presence of either a *Su(var)3-9* or *Su(z)12* mutation improved female viability (Additional file 1: Tables S1 and S2). This cooperation between the two chromatin systems is most compellingly highlighted by the improvement in female viability when the dose of both *Su(z)12* and *Su(var)2-5* is halved, an improvement that is more effective than when the dose of *Su(var)2-5* and *Su(var)3-9* are halved. The interaction results were similar with different alleles of PcG genes (Additional file 1: Table S2), and the trends tended to match the allele strength, suggesting the results are not from background interactions but are related to the mutations themselves.

The effects of the PcG/trxG genes on *Sxl* were further analyzed by qRT-PCR for mRNA levels and by *in situ* hybridization. Reducing the PcG/trxG genes affected both the strength and timing of the Sxl_Pe_ promoter (Additional file 1: Figure S8). We also determined the levels of H3K27me3 and H3K4me3 at Sxl_Pe_. H3K27me3 was generally lower in *ash1*^*MB03235*^/*TM6*, *Su(z)12*^*4*^/*TM6*, and *E(z)*^*731*^/*TM6* embryos, compared to wild-type (Additional file 1: Figure S7). Not unexpectedly, as they make up PRC2, *Su(z)12*^*4*^ and *E(z)*^*731*^ had the stronger effect.

These various analyses demonstrate that *Sxl* is a *bona fide* target of the PcG/trxG proteins despite the relatively low levels of their marks compared to more heavily modified genes such as *Ubx* and *hb*. We also note that *cta*, which has relatively low levels of H3K27me3 in embryos, shows significant levels of the mark in larvae as do *bnk* and *nullo*, supporting the notion that these genes are regulated by PRC2. The low and high levels of H3K27me3 at promoters additionally suggest at least two classes of targets, an idea consistent with the demonstration that genes such as *Polycomb*-*like*, which do not affect all PC targets [26], function not in the deposition of H3K27me3 but in elevating its levels at targets like *Ubx* which are highly modified*.* This potentially explains the two classes we observe.

### Promoters in early *Drosophila* embryos are bivalent

The gene promoters we analyzed had detectable levels of both H3K27me3 and H3K4me3 (Figure 2), suggesting they may be bivalent. Alternatively, the dual signals might be from the contribution of mixed tissues of expressing and non-expressing cells - the prevailing view that bivalent domains do not exist in *Drosophila* [27]. In the case of *Sxl*, the apparent bivalency would be from males having the repressive H3K27me3 and females the activating H3K4me3 mark. To determine whether the promoter is indeed bivalent, we analyzed an attached-X chromosome (X^X) stock (Additional file 1: Figure S7C,D) where the free X chromosome has a deletion of *Sxl* (*Sxl*^*fP7BO*^). In this stock, only females have *Sxl* sequences so the signal is representative of only females. H3K27me3 levels at *Sxl* in this stock were relatively low, so most of the signal in wild-type embryos comes from males. However, both sexes have comparable levels of H3K4me3 (Additional file 1: Figure S7C; male signal calculated from wild-type by subtracting out the female contribution). As Sxl_Pe_ is never expressed in wild-type males [22,28], this data clearly demonstrates the promoter is first set up in the bivalent state. This bivalency would be analogous to promoters in mammalian ESC which keep developmentally regulated genes transcriptionally poised. These domains resolve into either repressive H3K27me3 or activating H3K4me3 on differentiation [29,5], as seen in females as Sxl_Pe_ is transcribed.

**Figure 5:**
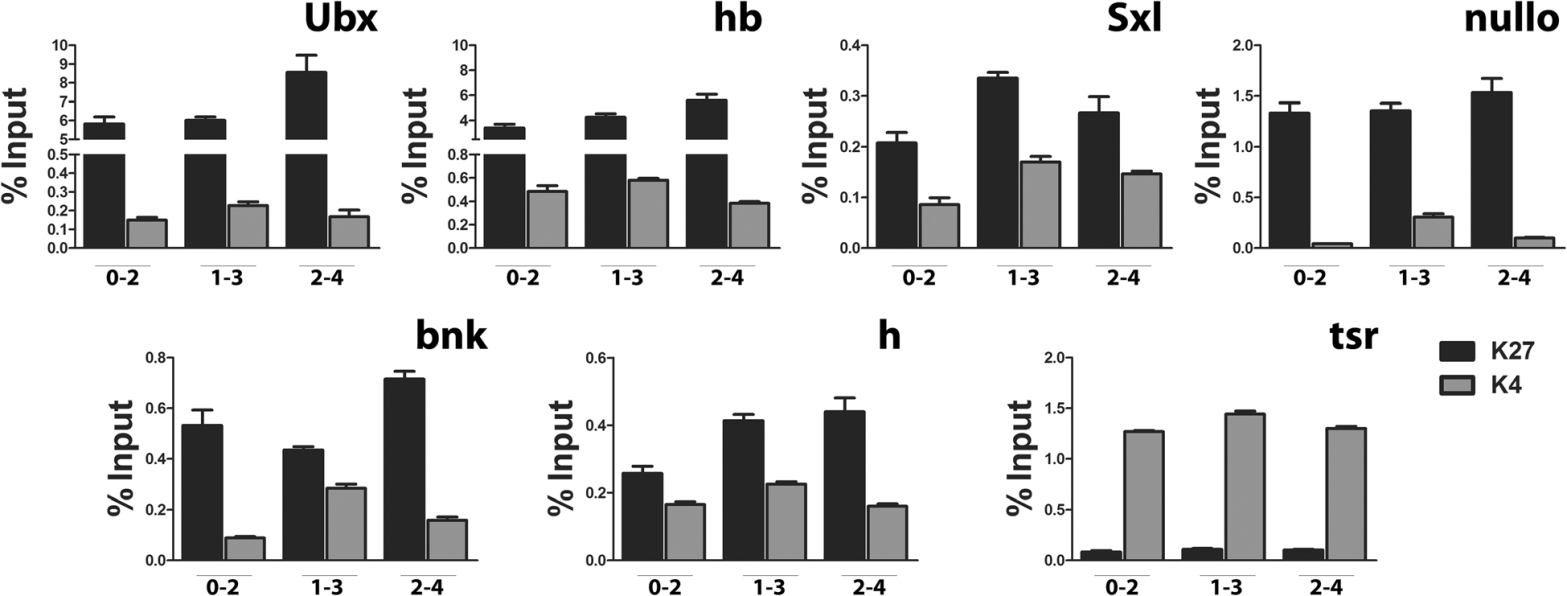
*Drosophila* gene promoters in early embryos are bivalently marked. ChIP data for H3K27me3 and H3K4me3 for *Ubx*, *hb*, *Sxl*, *nullo*, *bnk*, *h*, and *tsr*. Note scales for each gene. *Ubx* is bivalent in both 0–2 and 1–3 h embryos before it is transcribed; *hb* begins expression toward the end of the 0–2 h window. Sxl_Pe_ and *h* begin transcription in cycle 12 (approximately 110 min). *nullo* and *bnk* are expressed globally and begin transcription in cycle 11 (approximately 100 min). The large maternal deposit for *tsr* (actin depolymerizing protein cofilin) masks this promoter’s timing, although its mRNA increases at 2–4 h suggesting embryonic transcription. Cofilin is required globally and the *tsr* promoter has dual marks, albeit low H3K27me3 levels, in all 3 windows. (ModENCODE temporal expression data). Signal is above NI in all cases. Error bars represent ± SEM. Ubx, Ultrabithorax; hb, hunchback; Sxl, Sex-lethal; bnk, bottleneck; h, hairy; tsr, twinstar.

To determine if other *Drosophila* promoters are bivalent, six additional genes (*hb*, *h*, *Ubx*, *tsr*, *nullo*, and *bnk*) were scored (Figure 5). In all three windows examined (0–2, 1–3, and 2–4 h), the promoters had both histone marks. H3K4me3 levels were highest in the 1–3 h window. For H3K27me3, the levels differed by the gene, presumably as a function of its transcription status and tissue expression. This demonstrates the specificity of the ChIPs as each biological replicate gave a consistent but different signal for each gene. More relevant to bivalency, the dual marks are in 0–2 h embryos before the promoters are transcribed; *hb* which begins expression toward the end of this window is the closest call (Figure 5). Unlike previous reports [27], we examined very young embryos (0–2 h), before cellular blastoderm and the mid-blastula transition, while all the cells are still pluripotent (a situation more closely related to the ESC of mammals).

### HP1a and PcG proteins are in one complex

**Figure 6:**
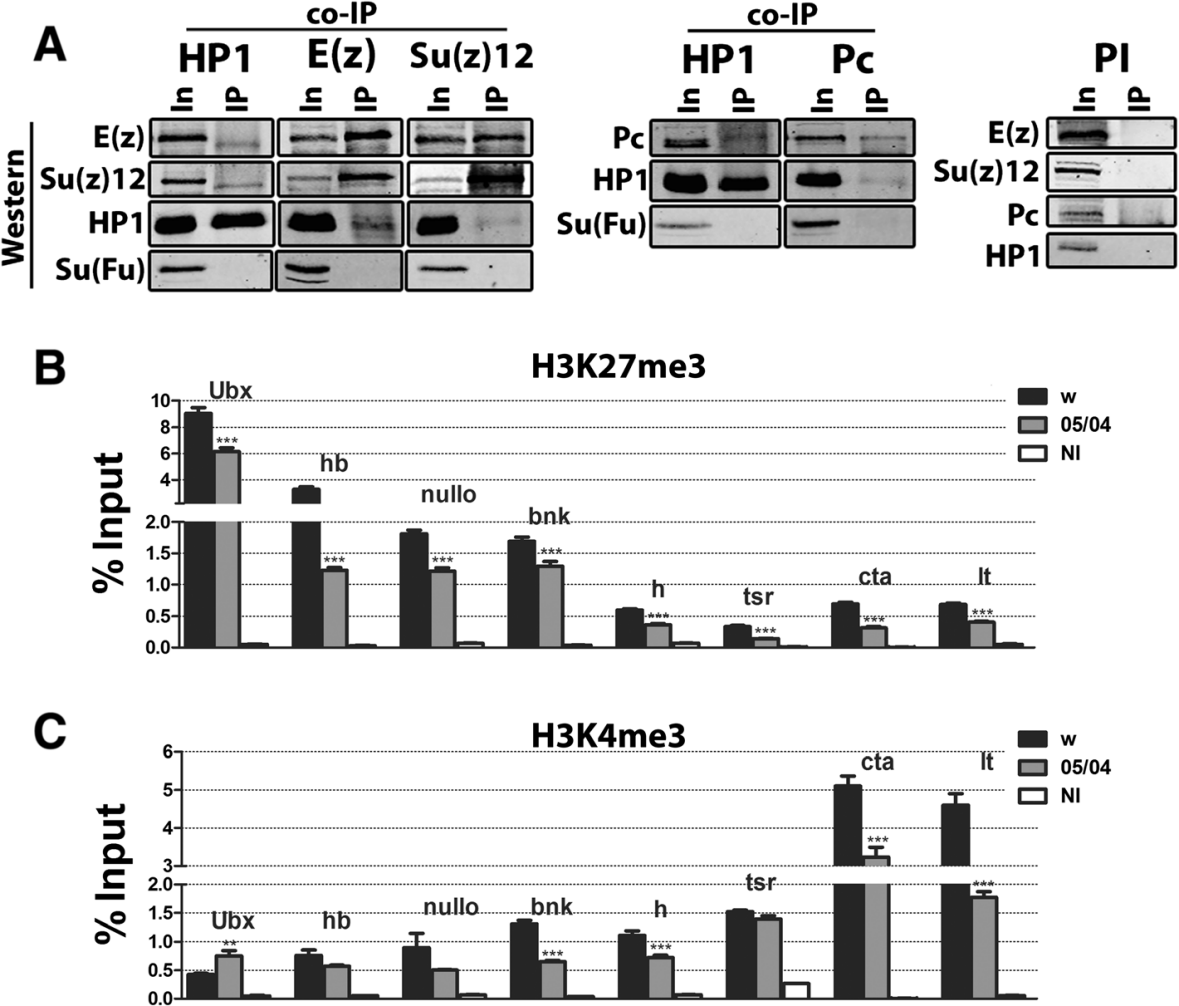
HP1a and PcG proteins are in one complex. **(A)** HP1a co-IPs with proteins in PRC1 and PRC2. IP antibody listed at top (left-right: HP1, E(Z), SU(Z)12, HP1 and PC) of the figure. For each IP 1% of the total Input (In) was loaded. One-third of the total IP loaded. Proteins detected with antibody listed on left of each panel set. Suppressor of Fused (SU(FU)) is the negative control. **(B, C)** HP1a is not pivotal for the maintenance of PcG/trxG marks. ChIP data from wild-type and *Su(var)2-5* ^*05/04*^ third instar larvae for H3K27me3 **(B)** and H3K4me3 **(C)** at *Ubx*, *hb*, *nullo*, *bnk*, *h, tsr, cta*, and *lt* promoters. All NI data is significantly below lowest ChIP signal. Asterisks show significant changes relative to wild-type, *P value <0.05, **P value <0.005, ***P value <0.0005. Error bars represent ± SEM. IP, immunoprecipitation; In, input; HP1, heterochromatin protein 1; E(Z), enhancer of zeste; Su(z)12, suppressor of zeste 12; Su(Fu), Suppressor of Fused; H3K27me3, trimethylated histone H3 at lysine 27; Sxl, Sex-lethal; Ubx, Ultrabithorax; hb, hunchback; tsr, twinstar; bnk, bottleneck; h, hairy; lt, light; cta, concertina; NI, non-immune.

The effect of reducing HP1a on the levels of H3K27me3 in early embryos and the genetic interactions between HP1a and the PcG/trxG proteins at *Sxl* and *Ubx* all indicate a link between the two chromatin systems, raising the question of how this occurs. In mammals, HP1α and SU(Z)12 have been shown to interact both *in vitro* and *in vivo* [30]. This data suggests a physical interaction between HP1a and PcG proteins, which we tested for in wild-type *Drosophila* embryos. Indeed, immunoprecipitation (IP) experiments on extracts from 1–3.5 h embryos (Figure 6A) show the PRC2 components (E(Z) and SU(Z)12) in addition to the PRC1 component PC, co-IP with HP1a. PRC1 and PRC2 have been described as co-occupying promoters in murine ESC [5]. In *Drosophila*, several independent analyses describe 200–400 genome-wide PC targets as being co-occupied by multiple PcG members [31,27,32], so this result is not unprecedented. When each of the PcG proteins is IP, HP1a can also be detected (Figure 6A). This is the first evidence of PcG components interacting with HP1a in *Drosophila*. As HP1a is present at all the promoters examined, these results explain how HP1a can influence the deposition of the marks by PcG/trxG proteins.

As HP1a is in complex with PRC2 and its reduction decreases H3K27me3 levels, we tested whether it also affected PRC2 recruitment. ChIPs for E(Z) were performed on 1–3 h embryos from wild-type and *Su(var)2-5*^*05*^ heterozygous parents. With the exception of the *lt* promoter which was unchanged, all the promoters examined show a decrease in E(Z) levels suggesting HP1a facilitates the recruitment of PRC2 to promoters (Additional file 1: Figure S9B). PRC2 recruitment to the PRE element in *bxd* (tre-2; Additional file 1: Figure S9B) was also negatively affected by the reduction in HP1a.

### HP1a has less of a role in the maintenance of PcG/trxG marks

As HP1a appears to be required for the deposition of both H3K27me3 and H3K4me3 at gene promoters in early embryos, we wished to determine whether this dependency persists later in development, where it is possible to further reduce the levels of HP1a using two defective alleles - *Su(var)2-5*^*05*^ and *Su(var)2-5*^*04*^, null and strong loss of function alleles, respectively. These larvae survive primarily on maternal HP1a until the third instar stage after which they die without pupating. ChIP-qPCR on wild-type (*w*^*1118*^, as the *Su(var)2-5* alleles are in this background) and *Su(var)2-5*^*05/04*^ larvae was performed. Validating our protocol and results, the H3K27me3 signal for the *Ubx* promoter in wild-type was similar to that in other studies [33]. Surprisingly, as the larvae have a much greater reduction in HP1a, the decrease in H3K27me3 as well as in H3K4me3 (Figure 6B) was not proportionally greater, and in some cases even less than that in embryos. For H3K4me3, the *hb*, *nullo*, and *tsr* promoters were slightly lower but not significantly different from wild-type while *Ubx* was actually a little higher in the *Su(var)2-5*^*05/04*^ larvae. The heterochromatic genes *lt* and *cta* showed a proportionately larger decrease in H3K4me3 than seen in embryos, consistent with the observation that HP1a is required for the robust expression of genes which naturally reside in heterochromatin (reviewed in [34]). The general failure, however, of the strong reduction in HP1a in larvae to further affect the levels of H3K27me3 and H3K4me3 would suggest that they are maintained independently of HP1a, presumably from starting levels established earlier in development (note the mothers are heterozygous for *Su(var)2-5*^*05*^ so the marks in the *Su(var)2-5*^*05/04*^ larvae should at first be comparable to embryos since both start development with approximately 50% maternal HP1a). The H3K27me3 levels in *Su(var)2-5*^*05/02*^ larvae by contrast showed even smaller differences from wild-type (Additional file 1: Figure S9A), suggesting that the chromodomain defective protein from the *Su(var)2-5*^*02*^ allele must provide enough activity to almost normalize this mark by the larval stage.

### Establishing H3K27me3 levels across the genome

**Figure 7:**
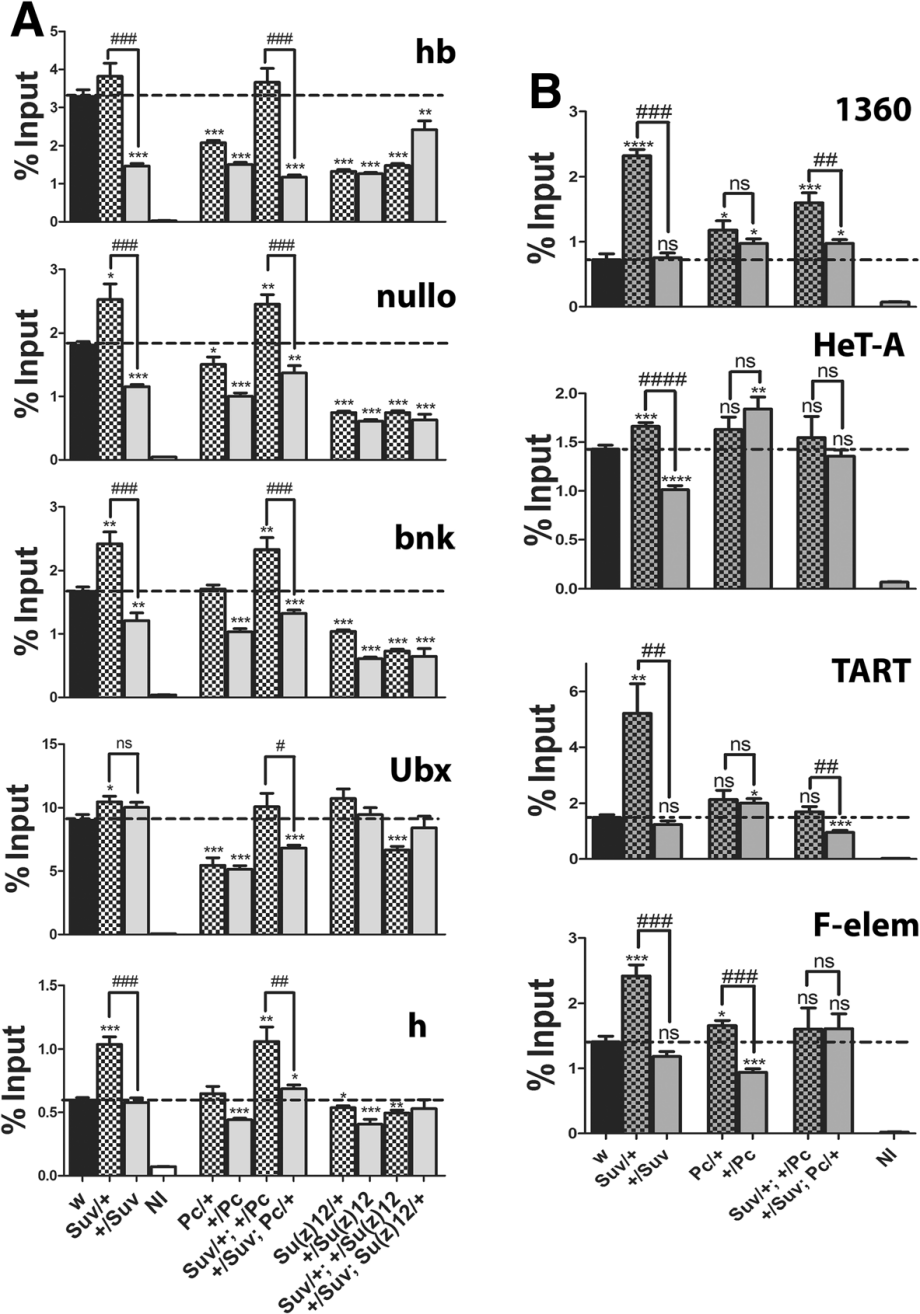
HP1a has a role in setting H3K27me3 levels. H3K27me3 ChIP data from wild-type, single or trans-heterozygous mutant third instar larvae. Mutation from the mother listed first (*/+), mutation from father listed second (+/*). The same applies for the double heterozygous condition. Strong loss of function or null alleles were used: *Su(var)2-5* ^*05*^, *Su(z)*12^4^, and *Pc* ^*15*^. **(A)** Promoters of silenced genes (*hb*, *nullo*, *bnk*) and active genes (*Ubx, h*); **(B)** heterochromatic regions (1360, HeT-A, TART and F-element). Broken line across is wild-type signal level. Asterisks show genotypes significantly different from wild-type and between the parental switch for *Su(var)2-5* and *Pc*; number symbol (#) shows significant differences between same genotype but the mutation is inherited from the opposite parent. *P value <0.05, **P value <0.005, ***P value <0.0005 (same applies for #). Error bars represent ± SEM. ns, not significant; hb, hunchback; bnk, bottleneck; Ubx, Ultrabithorax; h, hairy; NI, non-immune; Su(z)12, suppressor of zeste 12.

The strong embryonic effect on H3K27me3 by HP1a reduction implicated the genotype of the mother in establishing the mark. To test this and also determine how PcG mutations would influence the levels of H3K27me3, third instar larvae were generated for either *Su(var)2-5*^*05*^, *Pc*^*15*^ or *Su(z)12*^*4*^ where each mutation was inherited from a different parent. Additionally, trans-heterozygous mutants of *Su(var)2-5*^*05*^ with either *Pc*^*15*^ or *Su(z)12*^*4*^, where the parental origin of each mutation was reversed, were also generated. The three mutations are all strong loss of function, so they essentially reduce gene dose by half. H3K27me3 levels were measured at the promoters of three silenced (*hb*, *nullo*, and *bnk*) and two active genes (*Ubx* and *h*; Figure 7A).

The first point of note is that the parent which contributes the *Su(var)2-5*^*05*^ null allele affects the level of H3K27me3 detected in larvae. When the mother provides the mutation, H3K27me3 levels are actually higher than wild-type; however, when it is paternally inherited, they are lower. PC resembled HP1, where the maternal reduction showed lesser effects than the paternal (except for the highly modified *Ubx* gene which was equally reduced); however, unlike HP1, the levels did not exceed wild-type at any of the genes. In the case of *Su(z)12*, reducing its dose significantly reduced the H3K27me3 levels at silent genes regardless of the parent of origin, indicating that maintaining the silenced state is sensitive to the levels of PRC2 (Figure 7A). This is not entirely surprising, given that SU(Z)12 is part of the PRC2 enzymatic complex. For active genes, the reduction in *Su(z)12* had a rather modest effect and for *Ubx*, which normally contains high levels of H3K27me3, there appeared to be no effect. This suggests that unlike silenced genes, genes which are being transcribed can compensate for the reduction in SU(Z)12 or are less sensitive to its reduction.

The second interesting observation came from combining *Su(var)2-5*^*05*^ with *Pc*^*15*^. The levels of H3K27me3 were generally higher than when PC alone is reduced, and for *all* the genes the parent-of-origin effect on H3K27me3 recapitulated the *Su(var)2-5*^*05*^ trend: higher than wild-type if maternally inherited, lower if paternally inherited (Figure 7A). Remarkably, in the presence of *Su(var)2-5*^*05*^, the *Pc*^*15*^ mutation is almost invisible, demonstrating that the HP1a dose is epistatic to that of PC, and once again places HP1a upstream of PC. A significant parent-of-origin effect was not observed when *Su(z)12*^*4*^ was combined with *Su(var)2-5*^*05*^; the only exception was *hb* where the trend was the reverse of *Su(var)2-5*^*05*^ alone, and the levels did not exceed wild-type.

We note the larvae being compared are genetically identical and only the parental source of the mutation is different. This implies that the amount of HP1a the mother deposits into the egg influences the perceived ‘set point’ for H3K27me3 levels at promoters. To have a parental effect, defining this set point must be a relatively early decision, as ultimately the zygote must transcribe its own genes and the parental source of a mutation should no longer come into play.

### Heterochromatin formation marks an organization point

A key event which occurs early in development is the formation of well-organized constitutive heterochromatin, beginning shortly before and extending into cellular blastoderm [35]. This process involves significant recruitment of HP1a to the heterochromatin [36]. As maternal reductions in HP1a lead to an ‘overshoot’ in H3K27me3 levels at gene promoters by the larval stage, we hypothesized that the lower levels of maternal HP1a may trigger a different set point to the PcG genes as they generate H3K27me3. This change in set point may arise from H3K27me3 substituting for HP1a at heterochromatin. If this was the case, it might be expected that regions of constitutive heterochromatin might also show elevated levels of H3K27me3 in larvae. To test this, the different heterochromatic sequences - 1360 repetitive element, TART and HeT-A telomeric elements, and the F-element were examined (Figure 7B). Like the euchromatin promoters, all showed elevated H3K27me3 levels when the *Su(var)2-5*^*05*^mutation came from the mother, but were unaltered or lower (HeT-A) if the mutation came from the father, indicating a new H3K27me3 set point is also established for heterochromatic regions. Surprisingly, the *Pc*^*15*^ mutation tended toward slightly increasing H3K27me3 but, with the exception of the F-element, had no significant parent-of-origin effect (Figure 7B). Combining *Su(var)2-5*^*05*^and *Pc*^*15*^ showed the *Su(var)2-5*^*05*^ parent-of-origin effect was maintained for 1360 and TART but erased for HeT-A and F-element, indicating that the various heterochromatic regions are less uniform in their response than euchromatin.

**Figure 8:**
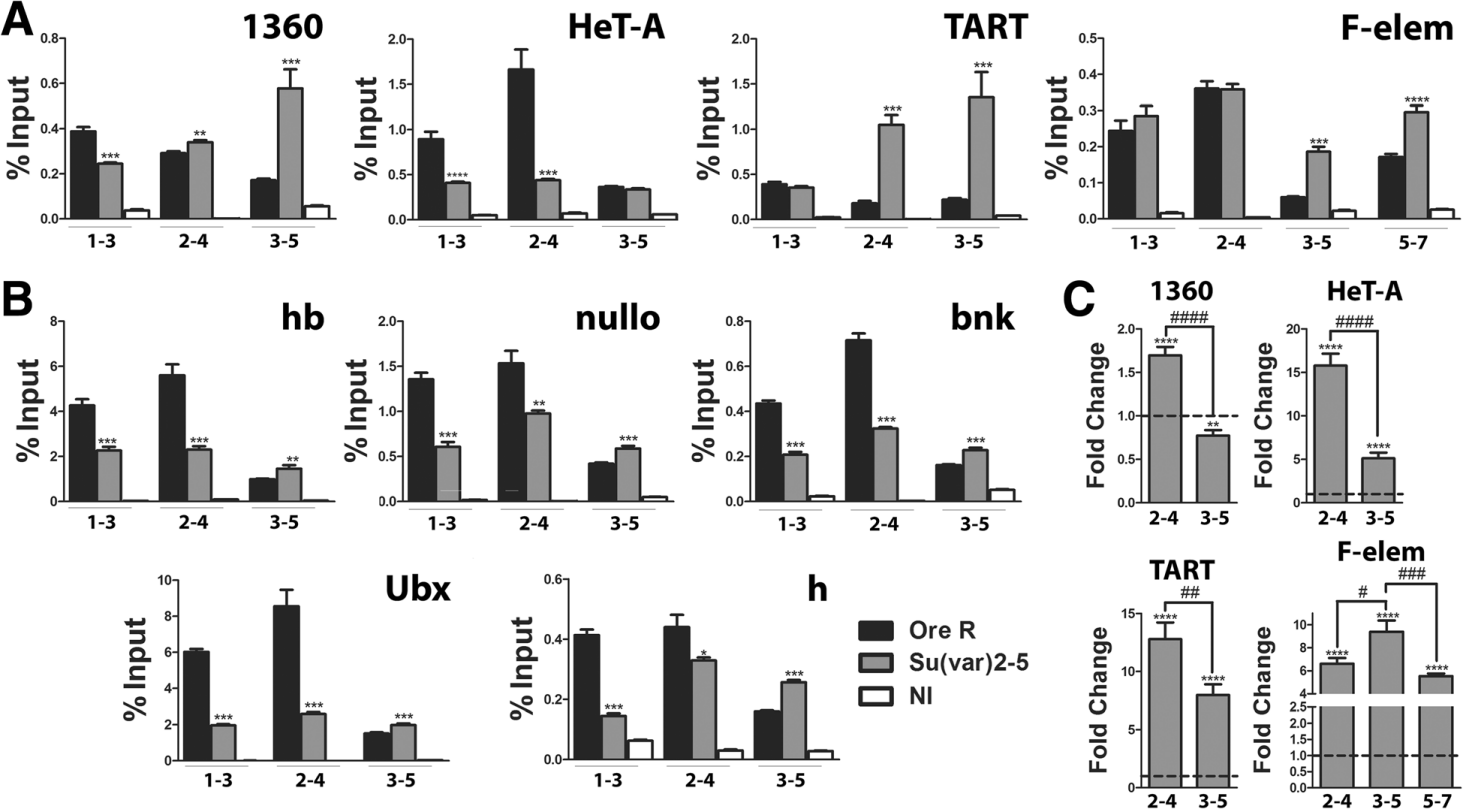
Cellular blastoderm and heterochromatin formation mark a key decision point for epigenetic marks. H3K27me3 levels in **(A)** heterochromatic regions and **(B)** gene promoters (*hb*, *nullo*, *bnk*, *Ubx*, *h)* during early development in wild-type and embryos from *Su(var)2-5* ^*05*^ */CyO* parents. Key to bar colors is in **(B). (B)** *hb*, *nullo*, *bnk*, *Ubx*, and *h* promoters scored during the time windows on the X axis*.* The signal at all the euchromatic gene promoters shows a switch between the 2–4 and 3–5 h windows going from less than, to greater than wild-type in the embryos from *Su(var)2-5* ^*05*^ */CyO* parents. The heterochromatic regions are a little more variable with TART and 1360 showing the switch a little earlier (2–4 h). Note the 1–3 h data is the same as in Figures 2A and 3, regraphed to show the changes over time. **(C)** Transcript levels of heterochromatic regions in 2–4 and 3–5 h embryos from *Su(var)2-5* ^*05*^ */CyO* parents relative to wild-type (set to one, broken line across). All but F-element show a decrease in the 3–5 window. F-element shows the decrease later in the 5–7 h window. Difference between time windows is significant in all cases. The RNA analysis takes into account the copy number of the repetitive elements (normalized to a single copy gene in both genotypes) as it has been previously demonstrated that loss of HP1a can alter the copy numbers of HeT-A and TART at telomeres [62,63]. Asterisks show differences significant from wild-type, *P value <0.05, **P value <0.005, ***P value <0.0005, ****P value <0.0001. Number symbols (#) in **(C)** are for the same *P* values. Error bars represent ± SEM. NI, non-immune; hb, hunchback; bnk, bottleneck; Ubx, Ultrabithorax; h, hairy.

To determine when in development this set point for H3K27me3 levels might be established, we examined the mark in embryos from *Su(var)2-5*^*05*^/CyO parents over sliding time windows (1–3, 2–4. and 3–5 h). In 1–3 h embryos, a window which precedes heterochromatin formation, promoters in embryos from *Su(var)2-5*^*05*^ heterozygous parents have lower than wild-type H3K27me3 levels (Figures 2A and 8B). By contrast, TART and F-elements have normal levels, while 1360 and HeT-A show reduced levels (Figure 8A). In the 2–4 h window, gene promoters still show a deficit relative to wild-type while the heterochromatic regions, except HeT-A, are higher than or equal to wild-type (Figure 8A,B). The 2–4 h window straddles the cellular blastoderm stage, when heterochromatin formation is completed. In 3–5 h embryos, which is beyond heterochromatin formation, not only are the repetitive elements higher than wild-type but so are the gene promoters. Taken together, these results suggest a transition occurs around the cellular blastoderm stage. Maternal reductions in HP1a at first compromise H3K27me3 levels, but after cellular blastoderm, the levels of the mark exceed wild-type at both gene promoters as well as heterochromatic sites. Paternal reductions, as analyzed in larvae, show less of an effect and they tend to be reductions not increases in H3K27me3 levels.

To determine whether the changes in H3K27me3 at heterochromatic regions have transcriptional consequences, the transcripts from 1360, TART, HeT-A, and F-element were measured in 2–4 and 3–5 h embryos, windows in which the H3K27me3 levels show a switch relative to wild-type. As shown in Figure 8C, the transcripts from these repetitive regions (corrected for copy number) are all elevated relative to wild-type (set to one) indicating they are derepressed. This is as expected as HP1a is required for silencing heterochromatin, and this effect is dominant. Between the mid-blastula transition window (2–4 h) and after cellularization (3–5 h) as the relative levels of H3K27me3 increase, three of the four regions show a decrease in transcripts suggesting the mark may serve to repress them. The only exception was F-element whose transcripts did not decrease, a consequence perhaps of it elevating its H3K27me3 levels rather late (3–5 h). Indeed, scoring F-element transcripts in 5–7 h embryos shows their levels are decreased (Figure 8A). Overall, these results suggest that the PcG proteins provide some compensation for the reduction in HP1a, through changing the levels of H3K27me3.

### H3K9me3 and H3K27me3 appear to ‘sense’ each other’s levels

The histone modification normally associated with HP1a is methylation of H3K9. This prompted us to determine whether the levels of H3K9 methylation also change when H3K27me3 levels are altered. The same regions were analyzed in embryos and larvae; the heterochromatin transition windows of 2–4 and 3–5 h in embryos from *Su(var)2-5*^*05*^ heterozygous parents and heterozygous *Su(var)2-5*^*05*^ larvae with different parents of origin. H3K9me3 was scored as it has been reported to track similarly to H3K9me2 [37].

**Figure 9:**
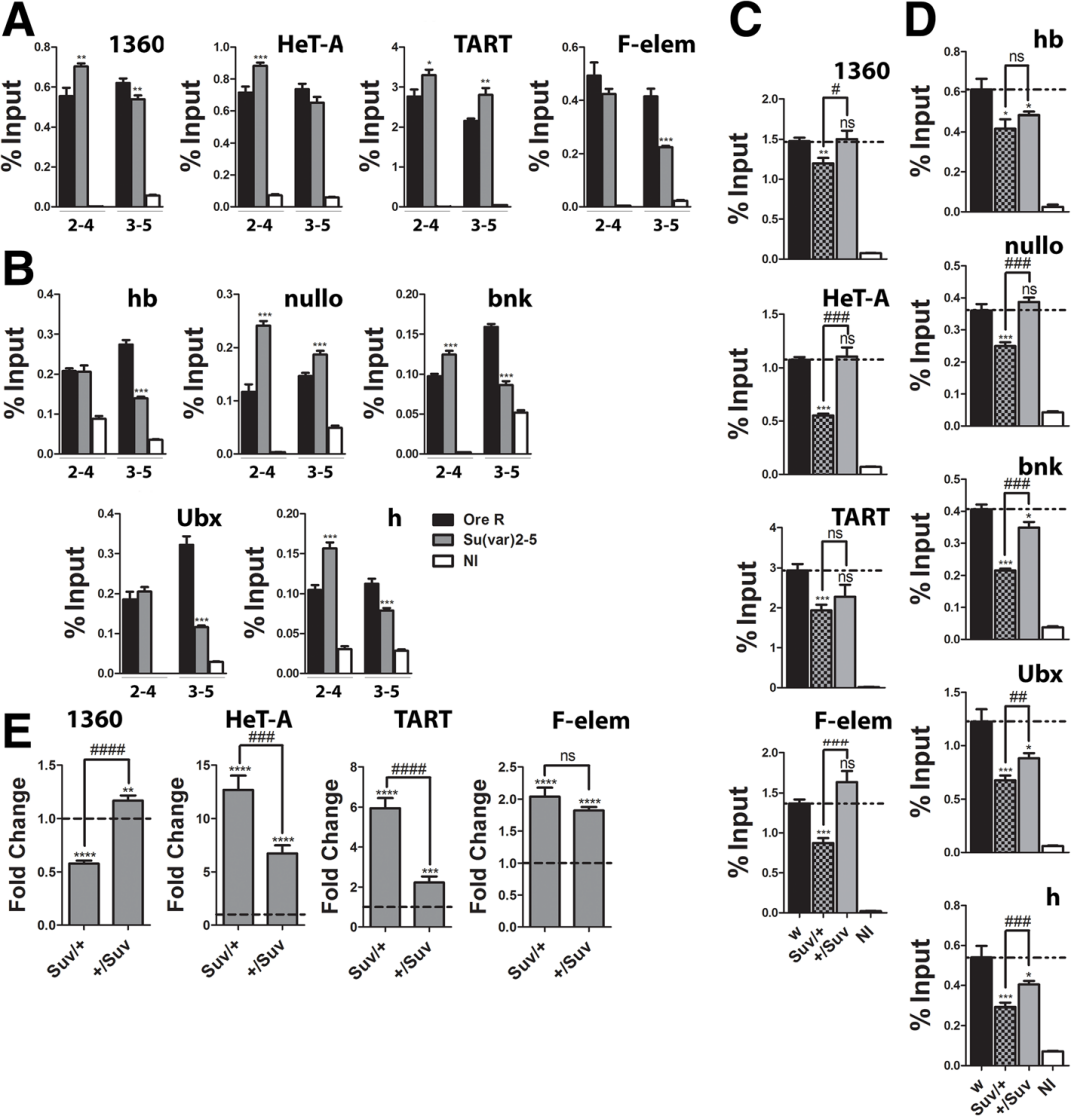
Reduction of HP1a also changes the amounts of H3K9me3, which generally are reciprocal to those in H3K27me3. H3K9me3 levels in **(A)** heterochromatic regions and **(B)** gene promoters (*hb*, *nullo*, *bnk*, *Ubx*, *h)* during early development (2–4 and 3–5 h) in wild-type and embryos from *Su(var)2-5* ^*05*^ */CyO* parents. Key to bar colors shown in **(B)**. Time windows on the X axis*.* Almost all regions, heterochromatic and euchromatic gene promoters, show a switch between the 2–4 and 3–5 h windows, going from greater than or equal to wild-type to less than wild-type in embryos from *Su(var)2-5* ^*05*^ */CyO* parents (HeT-A, TART, and *nullo* are the exceptions). **(C)** H3K9me3 levels at heterochromatic regions (1360, HeT-A, TART, and F-element) and **(D)** gene promoters (*hb*, *nullo*, *bnk*, *Ubx*, *h)* when *Su(var)2-5* ^*05*^ is inherited from a different parent. Mutation from the mother listed first (*/+), mutation from father listed second (+/*). Broken line across is wild-type signal level. Asterisks show genotypes significantly different from wild-type and the parental types for *Su(var)2-5* ^*05*^. **(E)** Heterochromatic region transcript levels in larvae from opposite *Su(var)2-5* ^*05*^ parent relative to wild-type (set to one, broken line). The RNA analysis takes into account the copy number of the repetitive elements (normalized to a single copy gene in both genotypes). All show an increase except 1360 when *Su(var)2-5* ^*05*^ is maternally inherited. All except F-element show a difference in level between the *Su(var)2-5* ^*05*^ parental origin. Asterisks show genotypes significantly different from wild-type and *Su(var)2-5* ^*05*^ containing progeny; (#) symbol shows significant differences between same genotype but when the mutation is inherited from the opposite parent. *P value <0.05, **P value <0.005, ***P value <0.0005 (same applies for #). Error bars represent ± SEM. NI, non-immune; ns, not significant; hb, hunchback; bnk, bottleneck; Ubx, Ultrabithorax; h, hairy.

Repetitive regions which are clearly transcriptionally derepressed (Figure 8C), surprisingly, have levels of H3K9me3 that are elevated or not significantly less than wild-type in 2–4 h embryos (Figure 9A). In this same window, H3K27me3 levels are also elevated, except at HeT-A (Figure 8A). This suggests that in early embryos, the decrease in HP1a itself, and not the silencing marks, may be more critical to silencing these regions. Both marks may be elevated (by an unknown mechanism) to reduce the derepression. In the 3–5 h window, after the formation of heterochromatin, we generally find H3K27me3 levels are elevated while the H3K9me3 levels show a tendency to be reduced. The elevated H3K27me3 levels in the 3–5 h window do correlate with reduced transcription of the repetitive elements (Figure 8C). Note: Quantifying the mRNA levels for the two major H3K9 methylases, *SetDB1* and *Su(var)3-9*, shows them to be slightly higher than wild-type in the 2–3 h window but close to wild-type in the 3–5 h window (Additional file 1: Figure S4B), so their expression level is unlikely to be responsible for the lower H3K9me3 levels in the 3–5 h window.

The reciprocity between the levels of H3K27me3 and H3K9me3 is better upheld at euchromatin gene promoters (Figure 9B). In the 2–4 h window, when H3K27me3 levels are lower than wild-type, the H3K9me3 levels are either the same as or higher than wild-type (this increase is potentially explained by the observation that HP1a normally functions to restrict SU(VAR) 3–9 to heterochromatin [38]). In the 3–5 h window after the formation of heterochromatin we see the converse, H3K27me3 levels elevate while those of H3K9me3 decrease (for all but the *nullo* promoter which remained above wild-type but proportionately less so than in the earlier time window).

The larval stages also show a pattern of reciprocity for the two silencing marks at both euchromatin gene promoters as well as the repetitive elements (Figure 9). When *Su(var)2-5*^*05*^ is inherited from the mother, H3K9me3 levels are significantly lower than wild-type at the heterochromatic repeat regions (Figure 9D); however, the H3K27me3 levels are much higher (Figure 7B). When the *Su(var)2-5*^*05*^ mutation is inherited from the father, there is no significant change in H3K9me3 levels compared to wild-type, as for H3K27me3 (HeT-A was the only exception with lower H3K27me3). Regardless of the parent of origin, inheriting one copy of *Su(var)2-5*^*05*^ derepresses the repetitive elements (Figure 9E; only exception is 1360 in maternally inherited *Su(var)2-5*^*05*^), suggesting that, as seen in embryos, these chromatin marks cannot fully compensate for the reduction in HP1a. For the euchromatin gene promoters, we generally also find that when H3K27me3 levels are higher (maternal *Su(var)2-5*^*05*^), H3K9me3 levels are lower and, conversely, when H3K27me3 levels are lower (paternal *Su(var)2-5*^*05*^), H3K9me3 levels are higher (Figure 9D). The results combined, particularly at euchromatin, suggest coordination between H3K9me3 and H3K27me3 with the two repressive/silencing marks compensating for each other when HP1a levels are reduced.

## Discussion

Orchestrating transcription and its shutdown requires an intricate interplay between transcription factors, chromatin, and RNAP. The role of transcription factors in regulating this process has been the focus for several decades, but recent interest now includes chromatin modifying enzymes and their modifications on histones. The additional question of how epigenetic states are maintained through the cycles of cell division has also received much attention.

Two prominent chromatin modifying systems are those of the PcG/trxG and H3K9/HP1. Conventionally they are viewed as functioning independently, the former serving to mostly regulate genes in euchromatin, particularly developmental genes. The HP1 system is frequently viewed in light of constitutive heterochromatin and in silencing genes, although there have been several studies showing HP1a can act both positively and negatively in gene expression (reviewed in [4,39]).

### Early in development HP1a is necessary for establishing normal levels of both H3K27me3 and H3K4me3 at promoters

Our data suggest that in early *Drosophila* embryos, HP1a and PcG/trxG proteins regulate gene expression in a collaborative manner. HP1a is needed for the normal deposition of both H3K27me3 and H3K4me3 at several promoters (Figure 2). At some of the promoters, these effects can exceed 50%, suggesting a strong dependency for HP1a given that its levels have only been reduced. All ten promoters of the genes we examined were affected, that is, unaffected genes were not omitted. Statistically, the odds of not finding an unaffected promoter by chance in ten attempts are extremely low, which suggests that most gene promoters in early embryos must have their H3K27me3 and H3K4me3 deposited through a mechanism involving HP1a. Additionally, while H3K27me3 levels decrease when the PRC2 components E(Z) and SU(Z)12 are reduced as expected (Figure 2), these PRC2 mutants do not systematically reduce H3K4me3 levels like HP1a (Additional file 1: Figure S3). These results clearly place HP1a upstream of the deposition of both marks. Indeed, reintroducing HP1 into the *Su(var)2-5*^*05*^*/CyO* background restored both H3K27me3 and H3K4me3 at most of the promoters we examined, supporting the conclusion that HP1a impacts the deposition of both marks (Additional file 1: Figure S4D,E).

The presence of both H3K27me3 and H3K4me3 at several promoters in 0–2 h embryos when very few zygotic genes are transcribed [40] suggests that early *Drosophila* embryos have bivalent promoters (Figure 5). This argument is best supported by the evidence from the sex determination gene promoter, Sxl_Pe_, where we took advantage of a stock which allowed assessing the levels of the two marks in only females, and to calculate the male contribution in the wild-type signal (Additional file 1: Figure S7C,D). These data indicate the promoter has both H3K27me3 and H3K4me3 in males, where it is never transcribed. Additional support for bivalency comes from the observation that both marks show a dependence on HP1a. HP1a is found at all the promoters and interacts with the members of PRC1 and PRC2. This interaction, with both the PcG and trxG complexes, presumably leads to the methylation of histone H3 at lysines 4 and 27. Consistent with this proposal, PRC1 and TRX-C proteins have been shown to co-occupy many gene promoters in *Drosophila* [41,42]. The deposition of H3K27me3 is dependent on PRC2 as well as PRC1 (Figure 7).

### Dual roles for HP1a

Given that HP1a affects both activating and repressive marks, it is perhaps not surprising that we find the same gene can show both a negative and positive response to changes in HP1a. The response depends on the degree of the reduction in HP1a (Figure 1). At most of the promoters we examined, HP1a appears to facilitate the recruitment, stability or pausing of RNAPII, affecting the amount of resident Ser-5P RNAPII (Figure 4). As several of the genes we examined show classes of embryos with reduced or elevated transcription (Figure 1B, Additional file 1: Figure S2), the reduced level of Ser-5P RNAPII at their promoters when HP1a is reduced may be indicative of destabilized or decreased pausing at the promoters, respectively. Many developmental genes, including all of those in Figure 1A, have been shown to have paused RNAPII at their promoters [43].

The binding of HP1a to promoters has been shown to be necessary to maintain an open chromatin structure [44]. If its role is to maintain paused Ser-5P RNAPII, its depletion potentially explains this observed loss of open chromatin. Reductions in negative elongation factor (NELF) similarly show a reduction in transcription, counterintuitive to the negative role of NELF, due to the loss in inhibition of nucleosome assembly in the promoter proximal region as RNAPII vacates the promoter [45,46]. So while reductions in HP1a may facilitate release of RNAPII from promoters and enhance transcription, its severe reduction may result in promoter closure, similar to NELF. HP1a is also bound to the promoters of active genes in heterochromatin (*Drosophila* chromosome 4 and pericentromeric regions), and this binding is independent of H3K9 methylation [47]. It is also found at many non-pericentric genes, and many of these genes are actively transcribed [48].

### Mechanism of PcG recruitment

A question which remains elusive is how PcG/trxG complexes are recruited to their target sites. As PRC2 promiscuously binds RNA, non-coding RNAs have been suggested to have a role and it has been proposed that they serve to recruit the complex to promoters [49,50]. We propose the HP1a at promoters provides a strong alternative mechanism (though not mutually exclusive). HP1a can bind to RNA, and RNAse treatment removes the protein from euchromatic sites and heterochromatin [51,52]. Additionally, HP1α, the mammalian ortholog of *Drosophila* HP1a, but not HP1β or HP1γ, directly interacts with SU(Z)12 *in vitro* through its chromoshadow domain and requires the PxVxL motif [30]. *Drosophila* HP1a co-IPs with PRC2 components, as well as PC (Figure 6A), and it recruits PRC2 to promoters (Additional file 1: Figure S9B). All the promoters we examined in embryos had HP1a and approximately 50%-60% of the *Drosophila* PcG binding sites genome wide, are reported to be associated with gene promoters [41].

A recent report using mouse ESCs also found that the histone methylase G9a together with its homologue G9a-like (GLP), could control PRC2 recruitment and H3K27 methylation at a subset (21.6% of 958) of common target genes [53]. Their ‘findings support a model through which G9a/GLP and PRC2 are co-recruited to some genomic loci, likely via a common targeting factor.’ We would submit this common targeting factor is HP1.

In heterochromatic regions, HP1a is recruited to promoters independently of methylation [47]. Our analysis of Sxl_Pe_ found that the telomeric protein HOAP made a substantial contribution to HP1a recruitment. Presumably, proteins such as HOAP work in combination with general transcription factors to delimit promoter regions as appropriate binding sites for HP1a. In mammalian ESC, many of the PcG target genes have been shown to be bound by three main pluripotency transcription factors, suggesting that at a subset of genes PcG proteins can collaborate with developmental transcription factors to repress transcription [5].

### Regulating the genome by the PcG and HP1 systems

The dual roles of HP1a as a repressor and activator are fairly well described, and we demonstrate additional evidence to that effect. At least some of this duality must arise from HP1a impacting the activities of the PcG/trxG genes. This role changes depending on the developmental stage. In the early *Drosophila* embryo, nuclei divide very rapidly and the genome is packaged with maternally provided histones which are primarily unmodified. We propose HP1a works with the PcG/trxG genes to maintain a silenced genome, while promoters are bivalently marked poised for expression. In this capacity HP1a is upstream of the PcG/trxG genes. Once PRC2 introduces the initial marks for repression, the dependency on HP1a becomes diminished as evidenced by the *Su(var)2-5*^*05/04*^ larvae, which fail to enhance the deficiency in H3K27me3 despite their highly reduced levels of HP1a [54]. There is ample evidence demonstrating that the E(Z) methylase in PRC2 needs to be stimulated for activity and H3K27me3 itself is a potent stimulator of the complex. In young embryos, HP1a (and proteins at the promoter, including G9a/GLP [53]) may provide the initiating catalyst for H3K27me3 deposition. Once introduced, the PcG presumably maintains the H3K27me3 through the cell cycles and becomes less dependent on HP1a.

We find that over development the H3K27me3 and H3K9me3 repressive systems are dynamic, coordinating their levels with each other at euchromatic regions as well as at repetitive elements where they appear to promote silencing when the regions are derepressed from a reduction in HP1a. Altogether, the picture which emerges is that the time around constitutive heterochromatin formation is a key decision point for setting the levels of the silencing marks. Although HP1a is upstream of the PcG/trxG initially, the silencing marks of the two systems appear to have reciprocity in their levels as the primary domains of their action, heterochromatin and euchromatin, respectively, are established.

In *Drosophila*, constitutive heterochromatin formation appears to complete around cellular blastoderm (approximately 3.5 h [35]). Across this time point (in 2–4 and 3–5 h embryos), embryos with reduced HP1a have lower than normal H3K27me3 signals but after constitutive heterochromatin has formed, the levels are higher than wild-type. By contrast, H3K9me3 levels are either higher or the same as wild-type in the early 2–4 h window and show a general reduction after completion of heterochromatin formation. Besides this apparent reciprocal nature in the levels of H3K27me3 and H3K9me3 across the heterochromatin formation point, it can also be observed when the parent of origin for the inheritance of the *Su(var)2-5*^*05*^mutation is reversed. Animals heterozygous for *Su(var)2-5*^*05*^ show different H3K27me3 and H3K9me3 amounts on their promoters, depending on which parent contributed the mutation. This parent-of-origin effect is generally reciprocal for both marks: whereas receiving *Su(var)2-5*^*05*^ maternally leads to higher H3K27me3 and lower H3K9me3 levels, receiving the mutation paternally leads to lower H3K27me3 levels and H3K9me3 levels that are frequently unchanged relative to wild-type, or higher than the maternally inherited *Su(var)2-5*^*05*^mutation.

**Figure 10:**
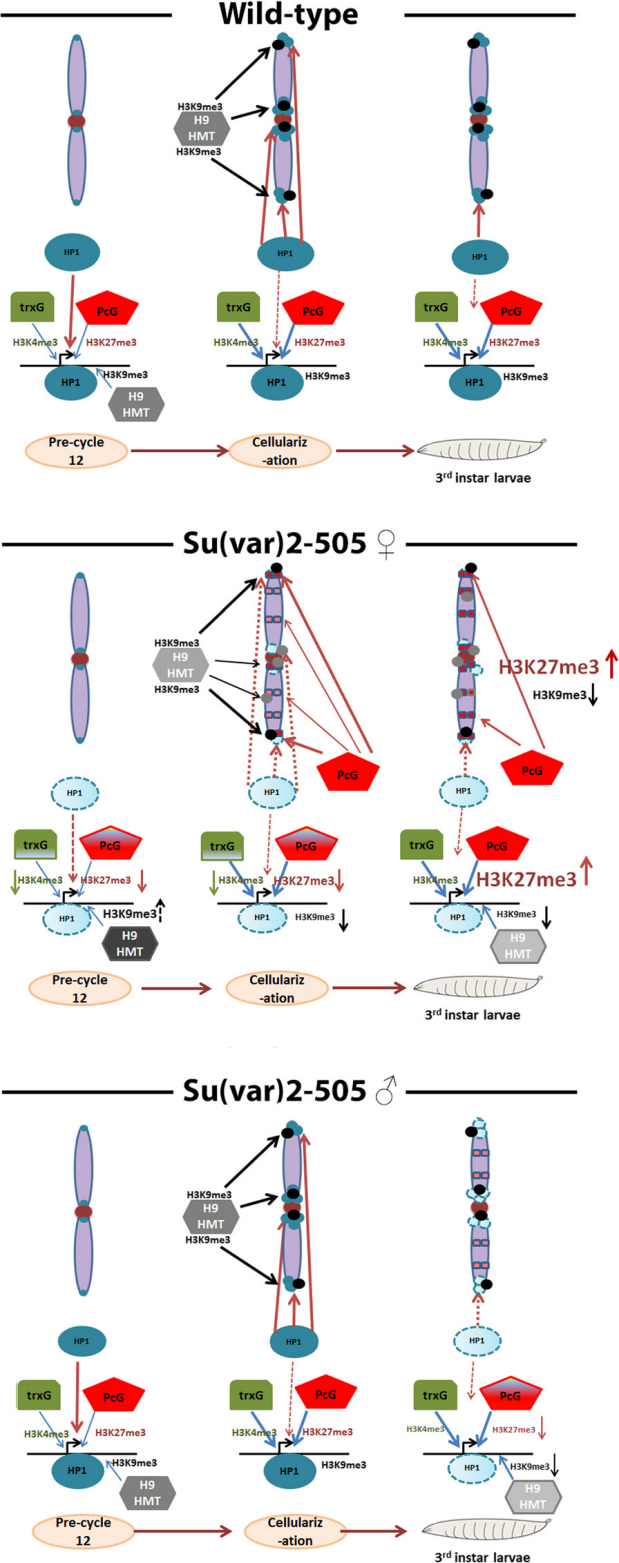
Model of interaction between HP1a and the PcG/trxG proteins. In early wild-type embryos, HP1a recruits the PcG/trxG proteins for H3K27me3/H3K4me3 deposition at promoters. H3K9me3 levels are also set. As constitutive heterochromatin is formed, HP1a is also recruited to these sites (blue circles), establishing the normal euchromatin promoter/heterochromatin distributions of PcG/trxG proteins and HP1a and their respective marks. These levels are maintained into the larval stage. When HP1a from the mother is reduced (middle), the embryo has reduced H3K27me3/H3K4me3 at promoters triggering an increase in H3K9me3 at promoters and heterochromatin (black dots). As constitutive heterochromatin forms, the insufficient maternal HP1a results in a derepression of repeated sequences. Pericentric H3K9me3 levels are reduced and telomeric regions are also affected. We propose this reduced HP1a stimulates the PcG chromatin system to compensate, elevating H3K27me3 levels. By third instar larvae, this causes an increase in H3K27me3 at promoters compared to wild-type, while the H3K9me3 levels are decreased. When the HP1a mutation is paternally received (bottom), maternal HP1a levels are normal in the early embryo. Constitutive heterochromatin can be formed normally without triggering the PcG chromatin system to compensate. All the silencing mark levels are presumed to be normal. However, as development proceeds, the levels of HP1a become insufficient due to the mutation, leading to a decrease in H3K27me3 as well as H3K9me3 at gene promoters. Repeated elements are less affected perhaps because their state was established early when maternal HP1a levels were normal. Size and weight of arrows, and color intensity depicts strength of effects or activity. K9 HMT depicts H3K9 histone methyltransferase (Setdb1 or Su(var)3-9 depending on the location). HP1, heterochromatin protein 1; H3K4me3, trimethylated histone H3 at lysine 4; H3K9me3, trimethylated histone H3 at lysine 9; H3K27me3, trimethylated histone H3 at lysine 27.

We speculate that when HP1a silencing is compromised, PRC2 becomes the back-up repressive mechanism. Indeed, the transcripts of repetitive regions show a decrease as the levels of H3K27me3 increase as embryos get older (Figure 8C). In early embryos (1–3 h), PRC2 is not the back-up repressive mechanism as HP1a is upstream of PRC2 at this stage. We find instead low levels of H3K27me3 at promoters and repetitive elements are depressed, both effects the outcome of reduced maternal HP1a (Additional file 1: Figure S4A). When heterochromatin formation begins, the situation is presumably further exacerbated, which then triggers the PcG system to contribute to the silencing of heterochromatin (modeled in Figure 10). We propose that at this stage in development the set point for H3K27me3 levels is established and embryos which have reduced maternal HP1a end up with their H3K27me3 levels at an elevated set point, at both heterochromatin and euchromatin, in the process of compensating for less efficient silencing by HP1a. The reduced HP1a level also affects H3K9me3, which in early embryos (1–3 h) responds to the low H3K27me3 and repetitive element derepression, and generally shows an increase. As H3K27me3 levels increase after heterochromatin formation, the H3K9me3 levels decrease.

This model also provides an explanation for the differences in levels of the silencing chromatin marks in the two parent-of-origin conditions. The foregoing explains the maternal inheritance. When the father introduces the *Su(var)2-5*^*05*^ allele, there is no reduction in the maternal supply of HP1a so the embryo can form constitutive heterochromatin normally. This does not trigger PRC2 to become the heterochromatin back-up system and the H3K27me3 set point is unchanged. The normal dependence for HP1a at promoters for the deposition of H3K27me3 is still present, however, and after formation of constitutive heterochromatin in the heterozygotes the reduction in HP1a is sensed (what constitutes the ‘sensing’ mechanism, remains to be determined). This results in a decrease in H3K27me3, much like early embryos with reduced HP1a before the formation of constitutive heterochromatin (Figure 10). This *Su(var)2-5* parent-of-origin effect dominates the effect of reducing the dose of PC, supporting the idea that it is HP1a which establishes the normal set point, and that its levels are more critical than those of PC.

Use of H3K27me3 as a back-up at constitutive heterochromatin is also seen during early mouse embryogenesis. For the first four divisions, the maternal chromosomes use H3K9me3 while the paternal chromosomes utilize PRC1 and H3K27me3. In the absence of maternal H3K9me3, PRC1 becomes the back-up repressive mechanism for the maternal chromosomes which then also use H3K27me3 to form pericentric heterochromatin [55].

The cooperation between HP1 and the PcG chromatin systems in regulating expression as well as partitioning the genome may be the offshoot of their having shared the same biological function. The chromodomains of HP1 and PC are very similar [56]. The mouse PC homologues (Cbx2, 4, 6, 7, and 8) do not all preferentially bind H3K27me3. Some show a preference for H3K9me3 while Cbx7 binds to both marks with affinities comparable to HP1 and PC [57]. In the ciliated protozoan *Tetrahymena thermophile*, the E(Z) homologue *EZL1* is responsible for both H3K27 and H3K9 methylation in RNA interference-mediated heterochromatin methylation. Both marks are recognized by the chromodomain protein Pdd1p [58]. In *Arabidopsis*, the HP1 homologue, like heterochromatin protein 1 (LHP1), binds to H3K27me3 in addition to H3K9me2/me3 and may regulate gene expression as part of the PcG silencing complex [59]. These studies indicate that at some point in evolution, the HP1 and PcG complexes had overlapping functions. The remnants of this interaction are presumably what we have uncovered during the window the *Drosophila* embryo organizes its genome into euchromatin and heterochromatin, to assign the primary roles of the two silencing systems between the two chromatin types.

## Conclusions

Our results demonstrate that HP1a and the PcG proteins work together to regulate both silencing and transcription. This interaction begins early in embryogenesis where HP1a appears to act upstream of the PcG/trxG marks. As the early embryo organizes its genome into heterochromatin and euchromatin, the HP1/H3K9me3 and PcG/H3K27me3 systems work together to both ‘sense’ and partition the genome, sorting the chromatin type each will regulate. In the event of reduced maternal HP1a levels, the PcG system serves as a back-up with an increase in H3K27me3 levels at heterochromatin, presumably to decrease the transcription of repetitive sequences which are derepressed by the reduction in HP1a. When H3K27me3 levels are low, particularly in euchromatin which is the primary domain of the PcG, there generally appears to be an increase in H3K9me3 levels. Our results suggest that at multiple steps/stages the two systems compensate for each other to collectively achieve the required silencing across the genome.

## Methods

### Crosses and transgenic lines

Flies were reared under uncrowded conditions on standard cornmeal medium. Description of genes can be found in Flybase (http://www.flybase.org/). All crosses were done at 25°C; *Ore R* and *w*^*1118*^ were the wild-type controls. Embryos from *Ore R*, *w*^*1118*^; *Su(var)2-5*^*05*^*/CyO*, *w*; *E(z)*^*731*^, *P{FRT(w*^*hs*^*)}2A TM6C*, *Sb*^*1*^, *Tb*^*1*^ or *w*; *Su(z)12*^*4*^,*e P{FRT(w*^*hs*^*)}2A/TM6C*, *Sb*^*1*^ parents were collected for 1 or 2 h on apple juice plates, aged appropriately for the RNA or ChIP collections, and processed as described. Larvae carrying the mutations of interest were sorted from the balancer class using the tubby marker on the third TM6B chromosome balancer for *Pc*^*15*^(w*; *P{hsp70-CD2.J}76 kni*^*ri-1*^*Pc*^*15*^*P{FRT(w*^*hs*^*)}2A/TM6B*, *Tb*^*1*^*)* and *Su(z)12*^*4*^, CyO-Kr-GFP was used for the two *Su(var)2-5* alleles (*Su(var)2-5*^*05/04*^) on the second chromosome. The RFP-HP1a transgene on the third chromosome (BL # 30562) was crossed into *w*^*1118*^; *Su(var)2-5*^*05*^*/CyO-GFP* background for the *Su(var)2-5*^*05*^ chromatin modification rescue; reduction of HP1 by RNAi used the transgenic TRiP line GL00531 (BL # 36792), the shRNA was driven by mothers with the maternal GAL4 *nos* promoter line 40 (BL # 4442). Genetic crosses testing female viability with mutant gene combinations had the progeny counted 8–9 days out from the first day of eclosion. All crosses had a minimum of 232 males, the reference class.

### *In situ* hybridization

*Hairy* and Sxl_Pe_*in situ* hybridizations were performed as described in [60]. *Ubx* and *bnk* probes were generated by primers listed in Additional file 1: Table S3. No obvious proliferation defects or signs of cell death as judged by DAPI staining were observed. This is not unexpected as the mothers are heterozygous for each mutation.

### qRT-PCR

RNA from appropriately staged embryos was extracted and quantified as in [60]. A minimum of two biological replicates was measured, each analyzed by three technical replicates for both the experimental and reference gene. mRNAs were quantified off an oligo-dT reverse transcription. Primers are listed in Additional file 1: Table S3. qPCRs were performed on a Bio-Rad CFX96 Real-Time PCR Detection System (Bio-Rad Laboratories, Inc., Hercules, CA, USA). C_q_ values which showed a difference of greater than 0.5 from the other two replicates were discarded. Statistical data analysis was completed using Microsoft Excel and GraphPad Prism.

### ChIPs

Chromatin was prepared as in [61]. For the E(Z) ChIPs, nuclei were fixed for 20 min instead of the standard 15 min. *Ore R* was the control. One-3 and 2–4 h collections used 80–100 mg of embryos. For the 0–2 h window, approximately 150 mg of embryos was used. ChIPs with larvae had approximately 35 third instars for each genotype. The only exception was *Su(var)2-5*^*05/04*^ where approximately 65 larvae were used. *w*^*1118*^ was the control. Larvae were first frozen at −80°C. To prepare the chromatin, they were first ground to a powder on dry ice using a mortar and pestle, then homogenized in nuclear isolation buffer (50 mM Hepes pH 7.6, 60 mM KCl, 250 mM Sucrose and 1x Protease Arrest (G-Biosciences, St Louis, MO, USA)). Subsequent steps were as described in Mulvey *et al.* [61].

ChIPs were performed using 0.24 ml clarified chromatin (20–40 μg anti-HP1, 3.5 μg of anti-H3K4me3 (Active Motif 39915, Carlsbad, CA, USA)), 2.5 μg anti-H3K27me3 (Active Motif 39155, Carlsbad, CA, USA), 3.5 μg anti-H3K9me3 (Active Motif 39161, Carlsbad, CA, USA) anti-Ser5-P (H14) (Covance MPY-127R, Emeryville, CA, USA), or non-immune serum (Sigma, St*.* Louis, MO, USA) with 30 μl of Protein A beads (Santa Cruz, Dallas, TX, USA) in 0.9 ml of RIPA buffer. For the anti-HP1 ChIPs, washes were performed as described in [15]. For the anti-H3K4me3 and anti-H3K27me3 antibodies, beads were incubated with the extract at 4°C, Wash Buffer 1 (500 mM NaCl, 10 mM Tris–HCl pH 8.0, 1 mM EDTA, 1% Triton-X 100, 0.1% SDS, 0.1% Sodium-Deoxycholate), Wash Buffer 2 (50 mM Tris–HCl pH 8.0, 2 mM EDTA, 500 mM NaCl, 1% NP-40, 0.1% SDS), Wash Buffer 3 (250 mM LiCl, 10 mM Tris–HCl pH 8.0, 1 mM EDTA, 0.5% NP-40, 0.5% Sodium-Deoxycholate) and TE (10 mM Tris–HCl pH 8.0, 1 mM EDTA). The IP material was then eluted.

ChIP quantification was performed as in [61]. As for RNA quantitation, a minimum of two biological replicates was scored, each with three technical replicates. C_q_ values that showed a difference of greater than 0.5 from the other two replicates were discarded.

Negative controls used NI serum (Sigma, St*.* Louis, MO, USA) and had at least two biological replicates.

### Immunoprecipitations

One-3.5 h *Ore R* embryo extracts from approximately 65 mg were homogenized in Buffer C Complete (100 mM Tris pH 8.0, 150 mM NaCl, 0.2% NP-40, 0.5% Triton-X 100, 1 mM DTT, 1x Protease Arrest). The whole cell extracts were pre-cleared in 5 μl Protein A/G beads for 1 h, diluted to approximately 0.6 ml and incubated with 2–9 μl of antibody for 2 h at RT and 2 h at 4°C. Antibody and extract were incubated with 5 μl of protein A or G beads for 2 h at RT and at 4°C overnight with rotation. IPs were rinsed and washed once for 5 min, then two more times for 10 min with rotation, each with 500-μl Buffer C. IPs were denatured and solubilized in 1x sample loading buffer, boiled, fractionated, blotted, and probed as described below.

Western blot experiments were performed by standard methods. Antibodies were used at: HP1 (1:7000 rabbit), (1:1000 mouse C1A9), E(Z) (1:600 rabbit), SU(Z)12 (1:500 rabbit), PC (1:500 rabbit), and Suppressor of Fused Su(Fu) (1:5 mouse). Blots were incubated with secondary antibodies (1:20,000 Li-COR Biosciences, Lincoln, NE, USA) and imaged by Odyssey Infrared Imaging System (Li-COR Biosciences, Lincoln, NE, USA).

## Additional file

Additional file 1:
**Supplementary figures and tables.** **Figure S1.** Interaction of HP1a and PcG/trxG chromatin systems. **Figure S2.** HP1a has dual effects on *bnk* transcription. **Figure S3.** Unlike *Su(var)2-5*, *Su(z)12* and *E(z)* do not consistently decrease H3K4me3. **Figure S4.** H3K27me3 and K3K4me3 reductions are from reduced HP1a and not from background effects or reductions in E(Z) levels. **Figure S5.** Global H3K27me3 levels do not show significant changes when HP1a is reduced. **Figure S6.** Non-immune (NI) controls of ChIPs in Figures 3 and 4. **Figure S7.** Evidence for PcG and trxG members in regulating *Sxl* from both genetic interaction data and chromatin modifications. **Figure S8.** PcG and trxG proteins regulate Sxl_Pe_. **Figure S9.** HP1a affects levels of PRC2 and H3K27me3 at promoters. **Table S1.** Female viability with decreased X chromosome counting genes in presence of PcG/trxG mutations alone and in combination with Su(var)2-5 or Su(var)3-9 mutation. **Table S2.** Additional PcG alleles tested for female viability effects. **Table S3.** Primers used in this study.

## Abbreviations

ash1: absent small and homeotic disks 1
bnk: bottleneck
ChIP: chromatin immunoprecipitation
cta: concertina
Esc: extra sex combs
ESC: embryonic stem cells
E(Z): Enhancer of zeste
GLP: G9a-like protein
H3K4me3: trimethylated histone H3 at lysine 4
H3K9me3: trimethylated histone H3 at lysine 9
H3K27me3: trimethylated histone H3 at lysine 27
h: hairy
hb: hunchback
HP1a: heterochromatin protein 1a
IP: immunoprecipitation
lt: light
NELF: negative elongation factor
NI: non-immune
PC: Polycomb
PcG/trxG: Polycomb group/trithorax group
PEV: position effect variegation
PRC1: Polycomb repressive complex 1
PRC2: Polycomb repressive complex 2
qPCR: quantitative PCR
RNAPII: RNA polymerase II
sgs8: salivary gland secretion 8
Sxl: Sex-lethal
Su(z)12: Suppressor of zeste 12
trx: Trithorax
tsr: twinstar
Ubx: Ultrabithorax
