# Supplementary material for: A balancing act: heterochromatin protein 1a and the Polycomb group coordinate their levels to silence chromatin in Drosophila
Source: Epigenetics Chromatin. 2015 Apr 30;8:17. doi: 10.1186/s13072-015-0010-z (PMC4423169; doi:10.1186/s13072-015-0010-z)
Supplement: Additional file 1: — Supplementary figures and tables. Figure S1. Interaction of HP1a and PcG/trxG chromatin systems. Figure S2. HP1a has dual effects on bnk transcription. Figure S3. Unlike Su(var)2-5, Su(z)12 and E(z) do not consistently decrease H3K4me3. Figure S4. H3K27me3 and K3K4me3 reductions are from reduced HP1a and not from background effects or reductions in E(Z) levels. Figure S5. Global H3K27me3 levels do not show significant changes when HP1a is reduced. Figure S6. Non-immune (NI) controls of ChIPs in Figures 3 and 4. Figure S7. Evidence for PcG and trxG members in regulating Sxl from both genetic interaction data and chromatin modifications. Figure S8. PcG and trxG proteins regulate SxlPe. Figure S9. HP1a affects levels of PRC2 and H3K27me3 at promoters. Table S1. Female viability with decreased X chromosome counting genes in presence of PcG/trxG mutations alone and in combination with Su(var)2-5 or Su(var)3-9 mutation. Table S2. Additional PcG alleles tested for female viability effects. Table S3. Primers used in this study. [file 13072_2015_10_MOESM1_ESM.doc]

**Supplemental Figures and Tables**

**Index**

Figure S1: Haltere phenotypes demonstrating genetic interactions between *Su(var)2-5* with PcG/ trxG genes. Related to Figures 1-4.

Figure S2: HP1a has dual effects on *bnk* expression. Related to Figure 1.

Figure S3: Unlike *Su(var)2-5*, mutations in *Su(z)12* and *E(z)* do not decrease H3K4me3 levels. Related to Figure 2.

Figure S4: Expression of HP1a is reduced in embryos from *Su(var)2-5^05^/CyO* parents and maternal expression of RFP-HP1a in same background. Expression of mRNAs of H3K27 and H3K9 methylases is elevated or unchanged in 2-3 and 3-5h embryos from *Su(var)2-5^05^/CyO* parents. E(Z) proteins levels also do not appear reduced. Reducing HP1a levels by RNAi produces a reduction in H3K27me3 levels, while introducing an HP1a transgene rescues most of the effects of the *Su(var)2-5^05^* mutation. Related to Figures 2 and 3.

Figure S5: Global H3K27me3 levels do not show significant changes when HP1a is reduced. Related to Figures 2 and S4.

Figure S6: ChIP negative controls. Related to Figures 4 and 5.

Figure S7: PcG and trxG members regulate *Sxl.* Evidence from both genetic interaction data and chromatin modifications. Related to Figures 1 and 2.

Figure S8: PcG and TrxG mutants affect *Sxl* expression. Related to Figures 1- 3 and 5.

Figure S9. HP1a affects levels of PRC2 and H3K27me3 at promoters. Related to Figures 3 and 5.

Table S1. Female viability with percentages and percent SE.

Table S2. Additional PcG alleles confirm interactions with Suvar genes is not a background effect.

Related to Table S1.

Table S3. Primers used in this study, Related to Figures 1-9.

**
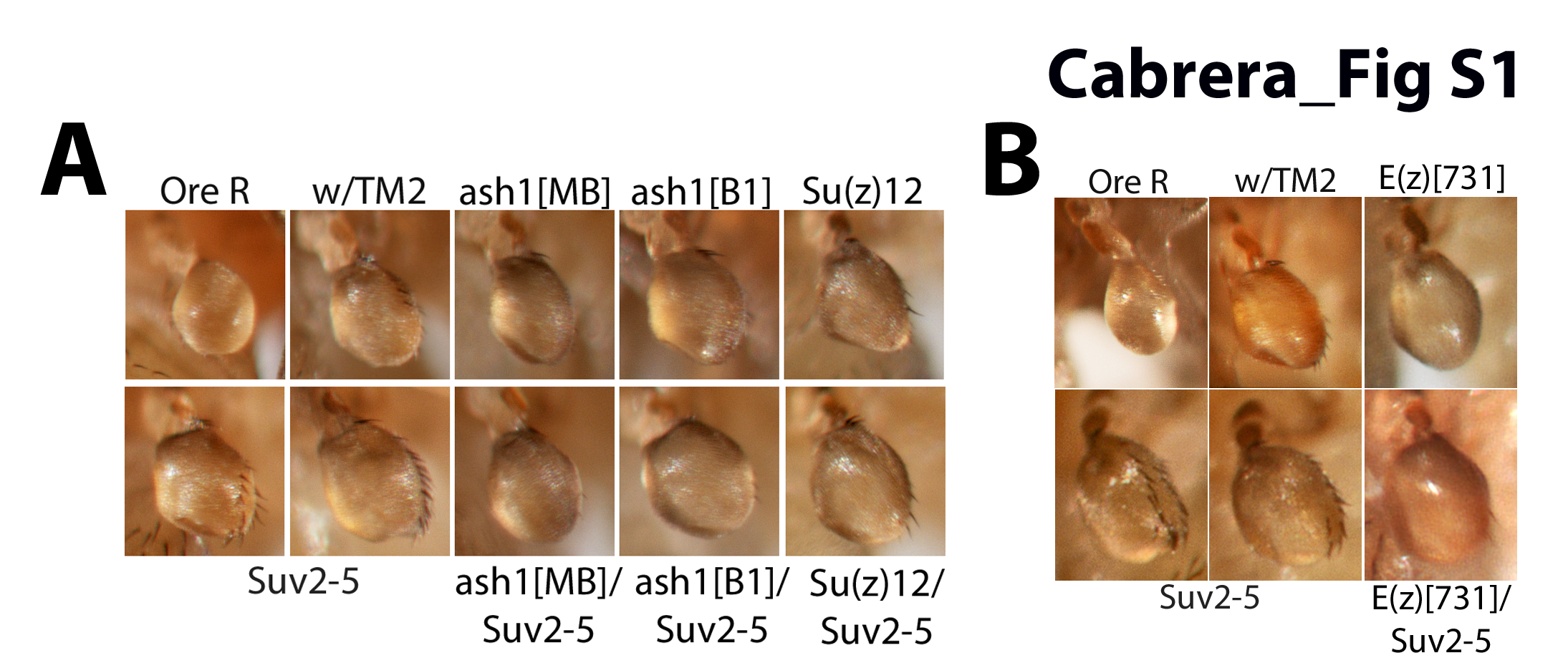
**

**Figure S1: Interaction of HP1a and PcG/trxG chromatin systems.** Changes in *Ubx^130^* haltere phenotype by presence of *Su(var)2-5^05^* and additional PcG/trxG mutations. Genotypes above and below pictures. Top rows show wild-type (*Ore R*), *Ubx^130^* (on TM2 balancer) or *Ubx^130^* with a single mutation. w is *w^1118^*. (A) Left 2 panels in second row show the effect of reducing HP1a dose using the null allele *Su(var)2-5^05^* (abbreviated Suv2-5). Note the enlargement, dual row of wing margin bristles. The rest of the images are *Ubx^130^* with double heterozygous mutant combinations. (B) Same interactions with *E(z)^731^* allele; images taken on different day with corresponding controls. Mutants which reduced the *Su(var)2-5^05^* haltere size also suppressed the dual row of wing margin bristles. *Su(z)12^4^* which remained enlarged also maintained the dual bristle rows, *E(z)^731^* alone and with *Su(var)2-5^05^* reduced the wing margin and double bristle rows but the size of the haltere remained somewhat enlarged. For reasons of space *ash1^MB03235^* is shortened to ash1[MB], *Su(z)12^4^* to Su(z)12.


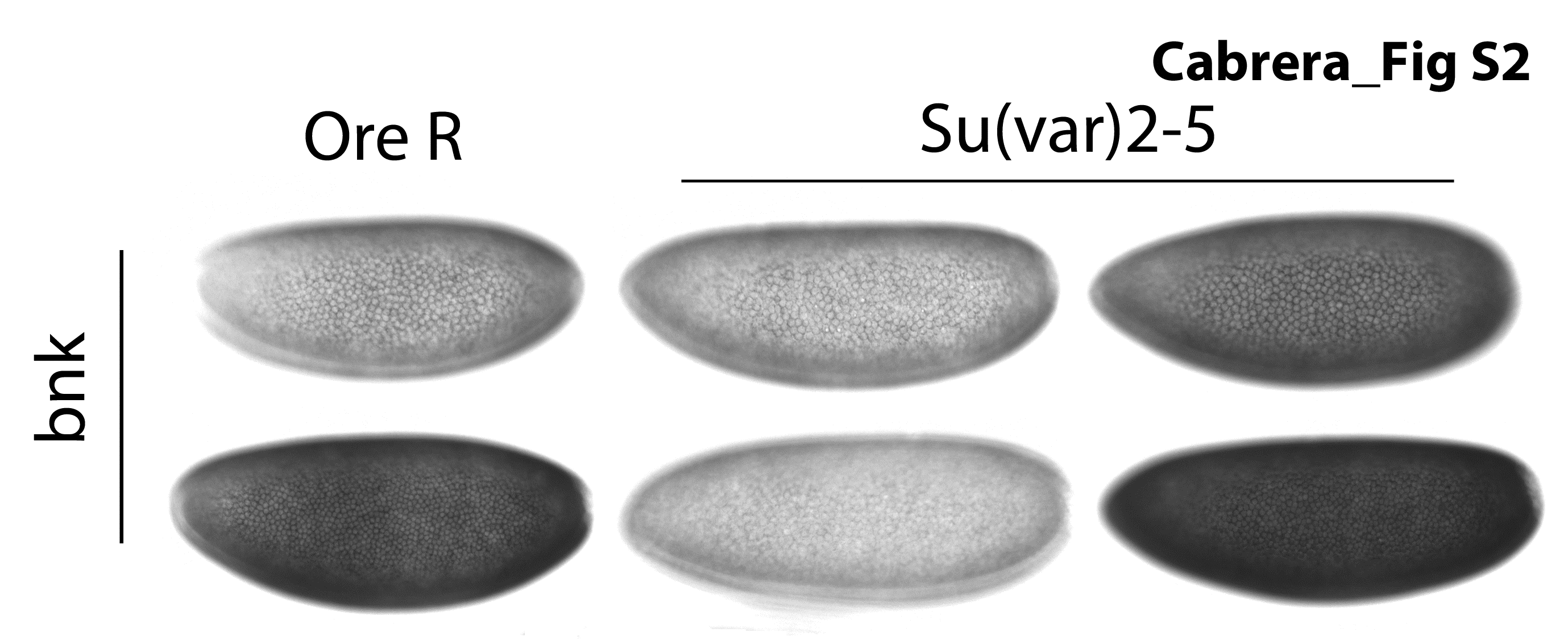


**Figure S2: HP1a has dual effects on *bnk* transcription.** *in situ* hybridization for *bnk* in embryos from *Ore R* or *Su(var)2-5^05^* heterozygous parents. Wild-type (*Ore R*) and *Su(var)2-5^05^* embryo images at same stage in the cell cycle shown. Weaker and stronger expression embryos could be detected in the *Su(var)2-5^05^* class. Top row cycle 13, bottom row cycle 14 embryos.


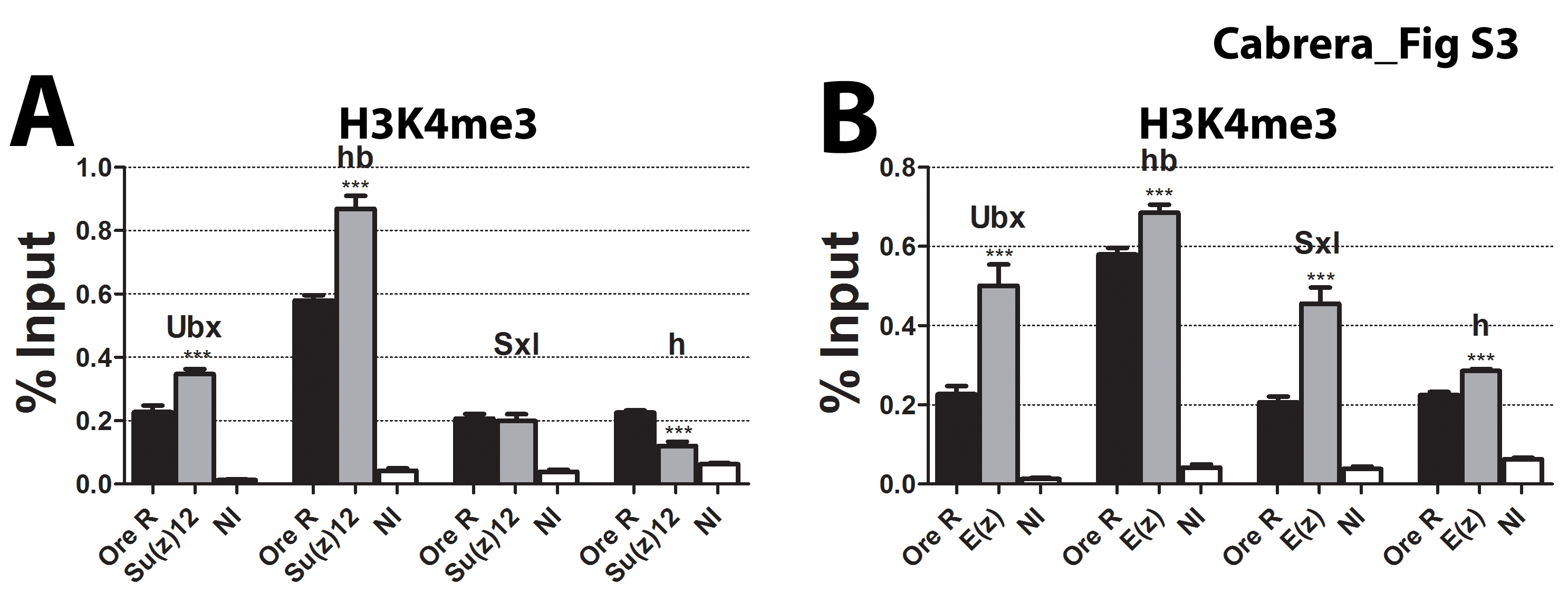


**Figure S3: Unlike** ***Su(var)2-5*, *Su(z)12* and *E(z)* do not consistently decrease H3K4me3.** (A, B) H3K4me3 ChIPs in 1-3h embryos from wild-type (*Ore R*) and *Su(z)12^4^/TM6* (A) or *E(z)^731^/TM6* (B) parents. H3K4me3 levels at the promoters of *Ubx*, *hunchback* (*hb*), *Sxl*, and *hairy* (*h*). Reducing *Su(z)12* and *E(z)* levels causes a general increase in H3K4me3, unlike the global decrease in H3K4me3 seen in embryos from *Su(var)2-5^05^/CyO* parents. NI ChIPs performed with non-immune serum. Error bars represent +/- SEM. Asterisks show significant changes relative to *Ore R* (*** p-value <0.0005).

**
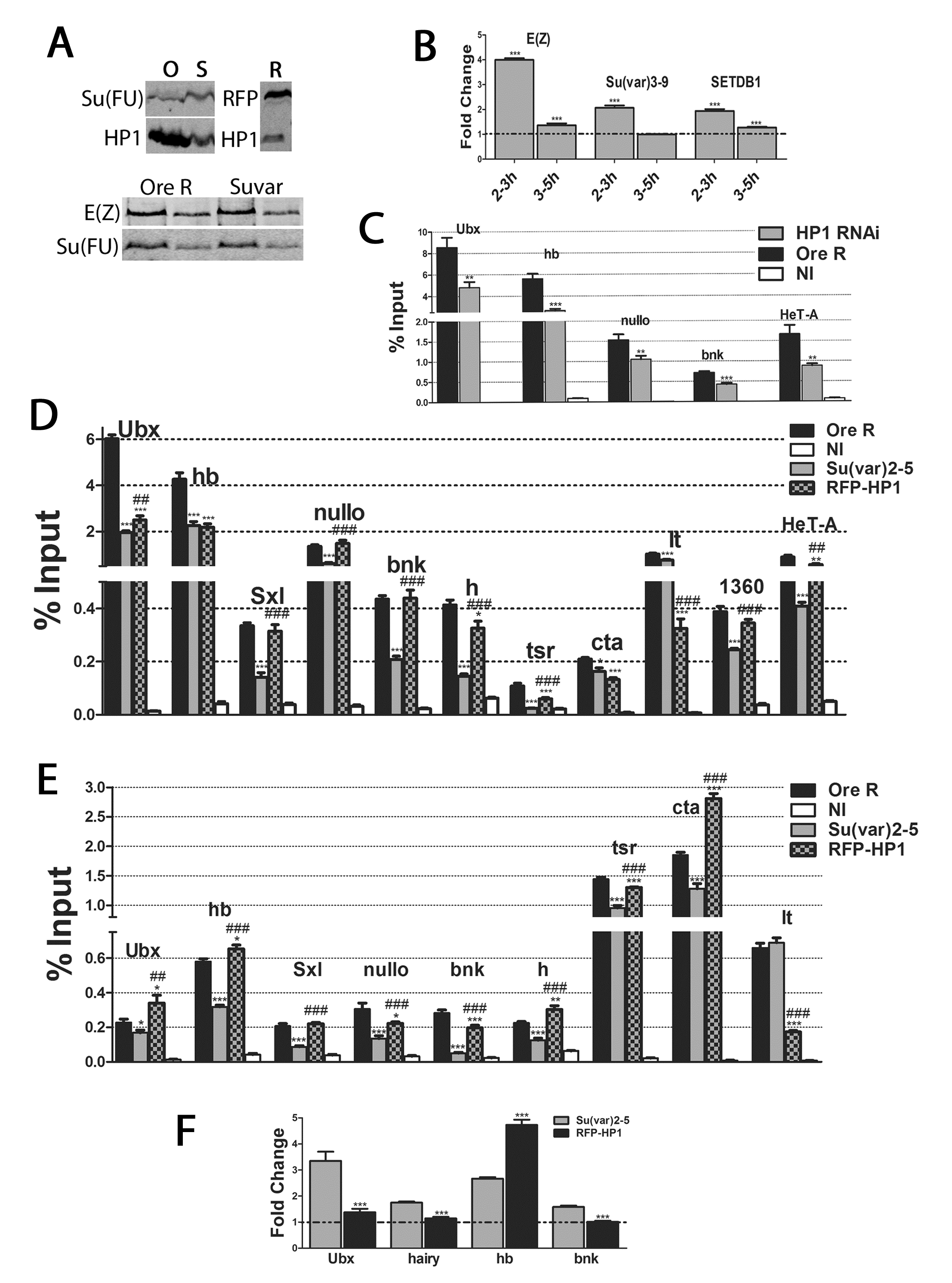
**

**Figure S4: H3K27me3 and K3K4me3 reductions are from reduced HP1a and not from background effects or reductions in E(Z) levels. (A) Maternal HP1a levels are reduced but E(Z) protein levels are normal in 1-2h embryos from *Su(var)2-5^05^/CyO* parents.** Top half shows Western blot probed for HP1a and Su(FU) protein. A second higher molecular weight band (R) is detected in the *Su(var)2-5^05^/CyO;* RFP-HP1a/+ background, indicating the presence of the RFP-HP1a fusion protein. Su(FU) protein serves as the loading control. Lower half shows *Ore R* and *Su(var)2-5^05^/CyO* (Suvar) 1-2h embryonic extracts probed for E(Z) and Su(FU) protein. Two dilutions of extracts loaded. **(B) mRNA levels of key methylases is not reduced in *Su(var)2-5^05^/CyO* embryos*.*** mRNAs of *E(z)*, *Su(var)3-9* and *SetDB1* were quantified by qRT-PCR in *Ore R* and *Su(var)2-5^05^/CyO* embryos before (2-3h) and after cellular blastoderm (3-5h). Levels from *Ore R* set to 1. **(C) Reducing HP1a levels by RNAi also produces a reduction in H3K27me3 levels**. To reduce embryonic HP1a levels maternal GAL4 using the *nos* promoter line 40 (BL # 4442) was used to drive expression of HP1 shRNA (TRiP line GL00531; BL # 36792). The H3K27me3 signal of 2-4h embryos is shown relative to wild-type embryos of the same age. Targets with the higher levels of H3K27me3 levels all show a reduction. Due to high background in 1-3h embryos slightly older embryos were analyzed. **(D, E) Introducing an RFP-HP1a transgene in *Su(var)2-5^05^/CyO* flies restores or improves both H3K27me3 (D) and H3K4me3 (E) levels.** The * symbol shows the statistical significance of *Su(var)2-5^05^/CyO* or the *RFP-HP1/+; Su(var)2-5^05^/CyO* lines compared to wild-type while the # symbol shows the statistical significance of *RFP-HP1/+; Su(var)2-5^05^/CyO* compared to *Su(var)2-5^05^/CyO*. **(F) The RFP-HP1a transgene in *Su(var)2-5^05^/CyO* flies can also ameliorate elevated transcription.** Expression levels of genes with elevated mRNA in Fig. 1A was measured in 2-3h embryos from *RFP-HP1/+; Su(var)2-5^05^/CyO* parents (RFP-HP1). *Ubx, h, bnk* mRNA levels decrease towards wild-type (set to 1), but *hb* continues to be elevated. Note: The RFP-HP1a transgene uses *Su(var)2-5*genomic sequences to drive expression but is unable to fully complement the *Su(var)2-5^05^*null mutation, perhaps because RFP blocks the amino terminus of HP1a. However, the RFP-HP1a transgene is maintained in the stock in the heterozygous condition without a balancer suggesting it is positive to the stock.  ^*^ p<0.05, ^**^ p<0.005, ^***^ p<0.0005. Error bars represent +/- SEM.


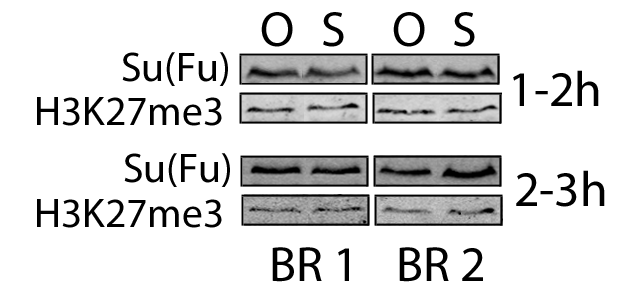


**Figure S5: Global H3K27me3 levels do not show significant changes when HP1a is reduced.** Western blots of H3K27me3 in embryos from wild-type (*Ore R* - O) and *Su(var)2-5^05^/CyO* (S) parents. Two different biological replicates (BR) for each time window shown; the age of the embryos is on the right. Loading reference is Su(FU) protein. Bands imaged and quantified using Li-COR Odyssey Infrared Imaging System (Li-COR Biosciences) technology; there is no significant difference in the marks after normalizing the signal to Su(FU) levels.


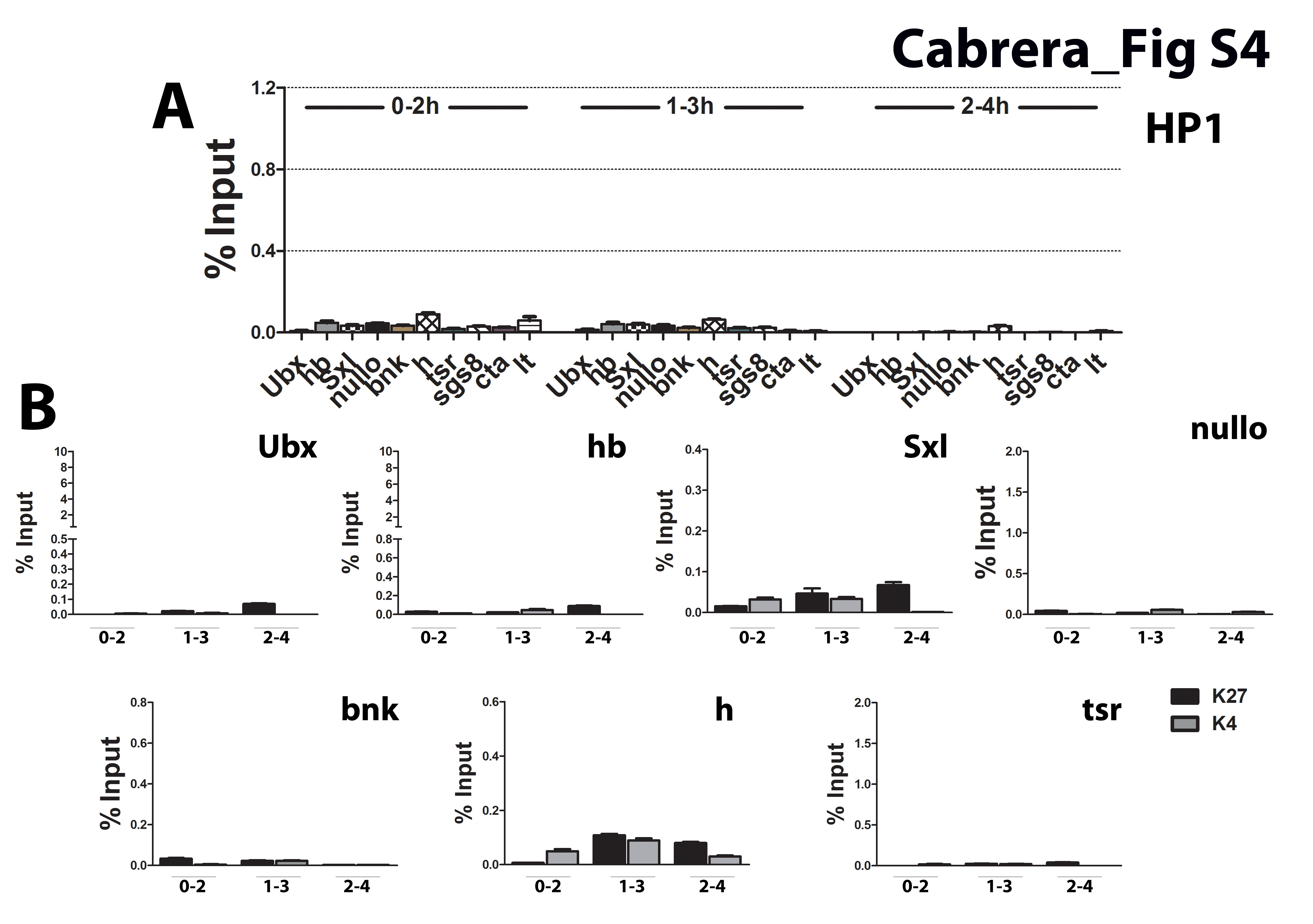


**Figure S6. Non-immune (NI) controls of ChIPs in Figs. 4 and 5.** Note for each graph the scale is identical to its corresponding panel and indicates much lower signal for the NI serum.


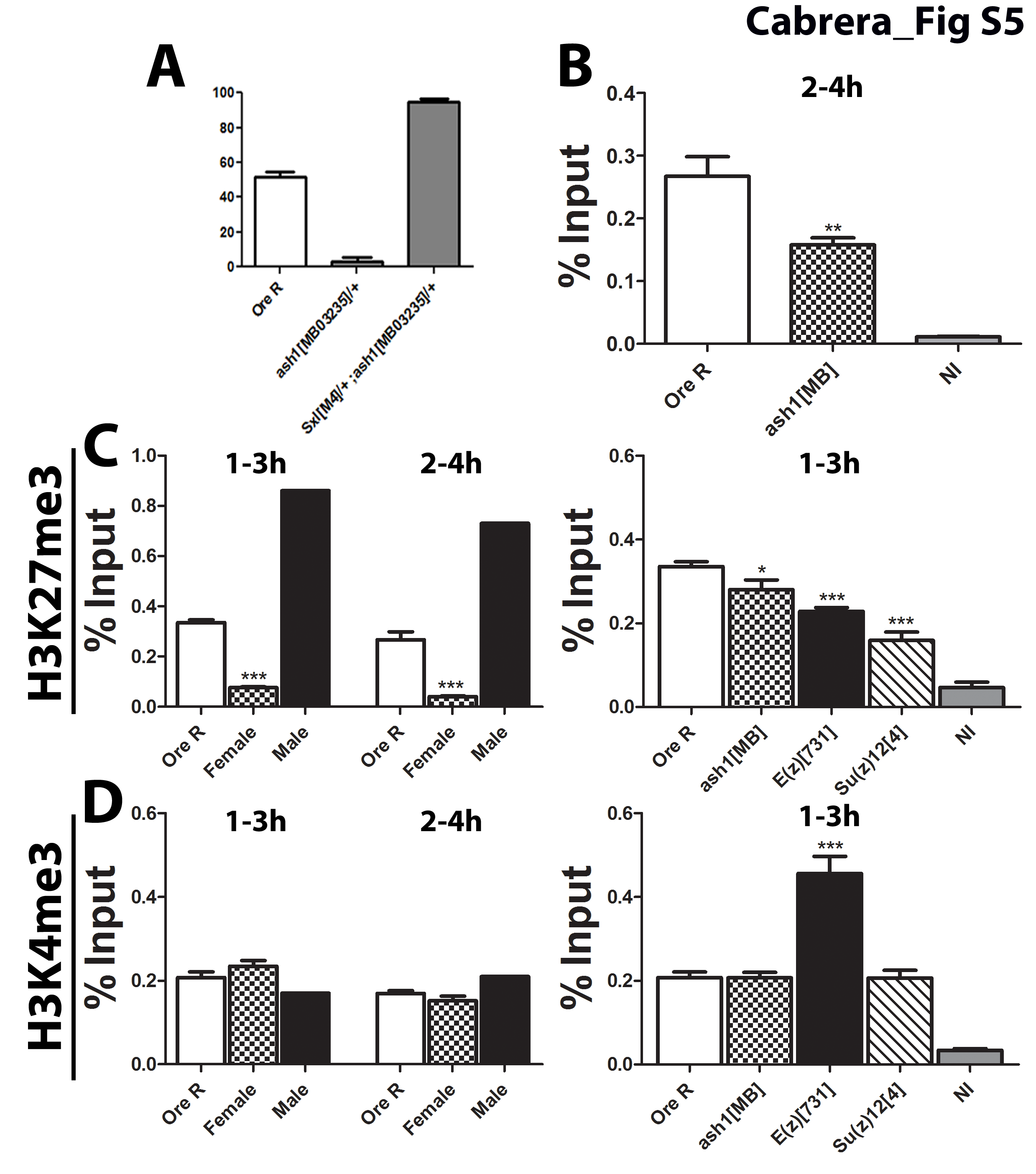


**Figure S7: Evidence for PcG and trxG members in regulating *Sxl* from both genetic interaction data and chromatin modifications.** (A) Percent female viability of *ash1^MB03235^/TM6* mothers and *ash1^MB03235^* with a constitutive allele of *Sxl*, *Sxl^M4^*, mothers crossed to males with reduced X chromosome counting genes. Presence of the constitutive *Sxl* allele completely rescues the female lethality. Note similar female lethality was observed for a second allele, *ash1 ^B1^*. Error bars represent percent SE. The wild-type (*Ore R*) control cross was performed in parallel with these crosses; food conditions were slightly different giving a higher baseline than in Tables S1 and S2. (B) H3K4me3 ChIPs in 2-4h embryos from wild-type (*Ore R*) and *ash1^MB^/TM6* parents at *Sxl*. Data represented as percent input. NI ChIPs performed with non-immune serum. **(C, D) PcG and trxG mutants change H3K27me3 and H3K4me3 levels at Sxl_Pe_.** ChIPs at Sxl_Pe_ (+138) using 1-3h and 2-4h embryos from wild-type (*Ore R*), *ash1^MB03235^/TM6*, *Su(z)12^4^/TM6*, *E(z)^7310^/TM6* and *X^X; Sxl^fP7B0^/Y* (Female) parents. Male ChIP data are estimates calculated from the *Ore R* and *X^X; Sxl^fP7B0^/Y* female data. Data represented as percent input. NI ChIPs performed with non-immune serum. Asterisks show significant changes relative to *Ore R* ^*^ p<0.05, ^**^ p<0.005, ^***^ p<0.0005. Error bars represent +/- SEM. Largest non-immune p-value comparison is <0.005. (C) H3K27me3, (D) H3K4me3.

**
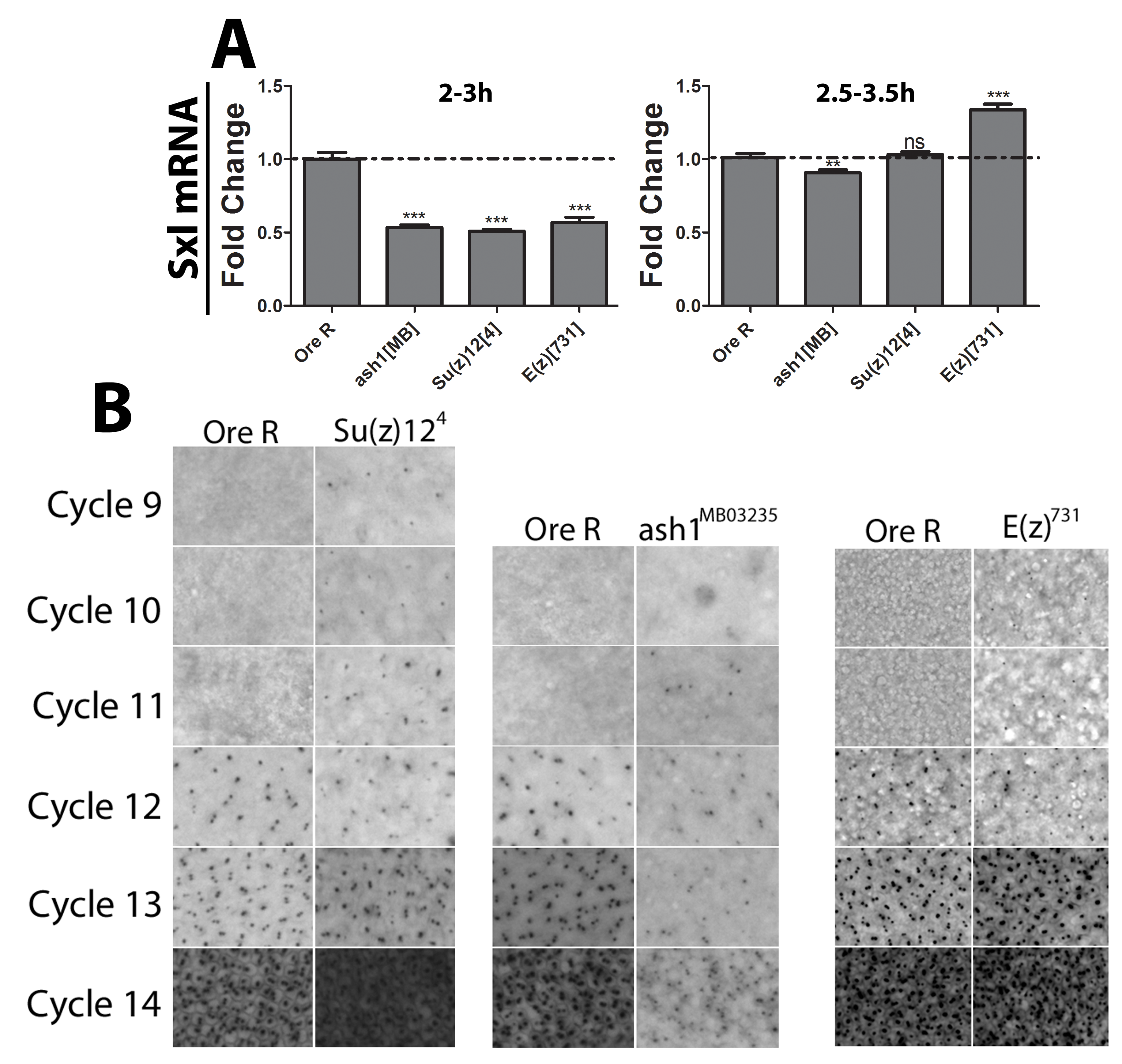
**

**Figure S8: PcG and trxG proteins regulate Sxl_Pe_.** (A) qRT-PCR measuring Sxl_Pe_ mRNA expression in 2-3h and 2.5-3.5h embryos from *Ore R*, *ash1^MB03235^/TM6*, *Su(z)12^4^/TM6*, and *E(z)^731^/TM6* parents. Two or more biological replicates for each genotype were analyzed. ns not significant, ^**^ p<0.005, ^***^ p<0.0005. Error bars represent +/- SEM. (B) *in situ* hybridization for Sxl_Pe_ specific transcripts in embryos from wild-type (*Ore R*), *Su(z)12^4^/TM6*, *ash1^MB03235^/TM6*, and *E(z)^731^/TM6* parents. Same sized areas shown of images taken at 40x. Mutant embryos displayed with corresponding *Ore R* embryos that were simultaneously processed. Nuclear cycle of embryos (designated by divisions) shown on left. In wild-type embryos a distinct signal is seen in only cycles 12-14. *Sxl* is on the X chromosome so females have 2 signal dots.

**
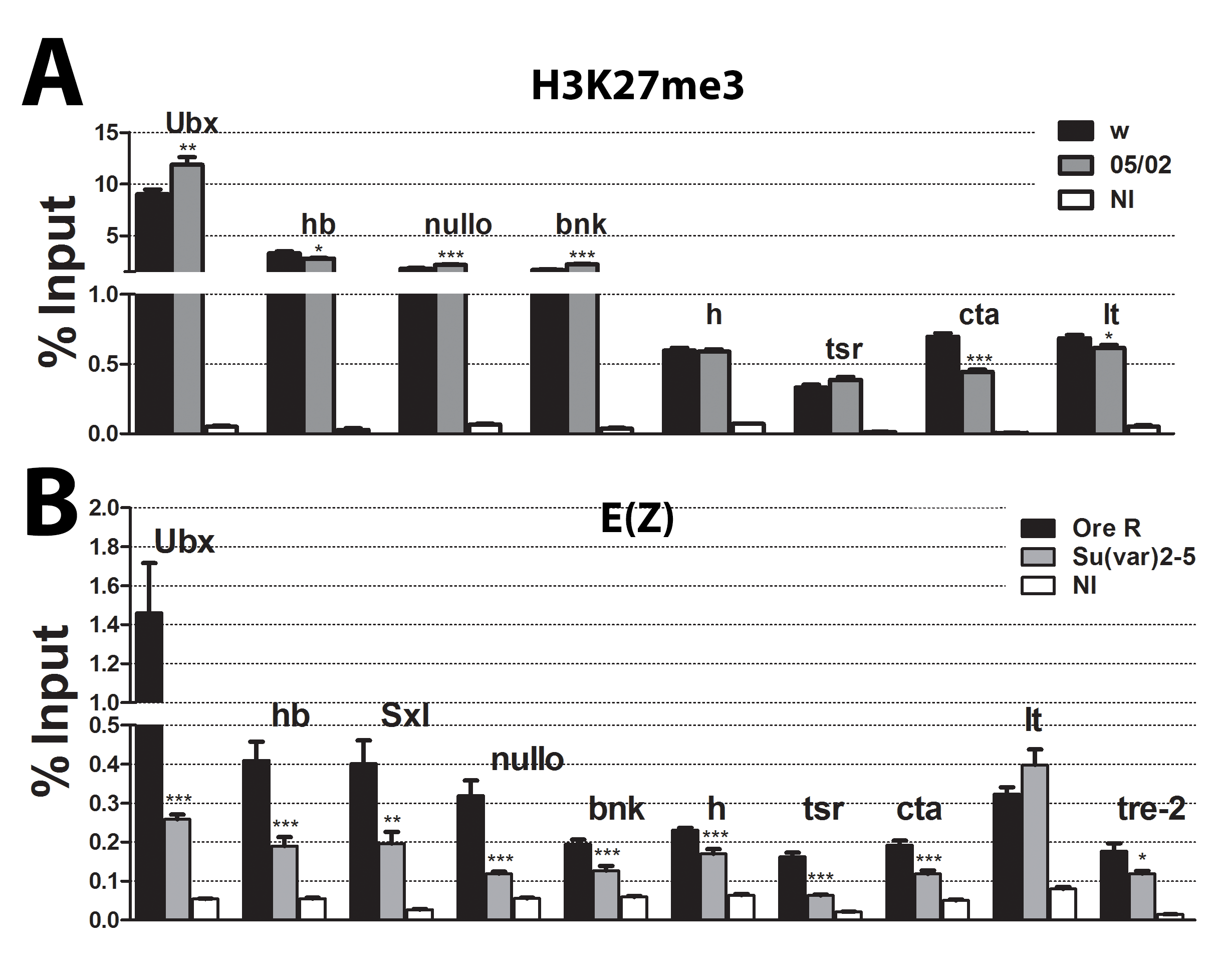
**

**Figure S9:** **HP1a affects levels of PRC2 and H3K27me3 at promoters.** (A) **H3K27me3 levels in *Su(var)2-5^05/02^* mutant larvae.** H3K27me3 ChIP data from wild-type (*w^1118^*) and *Su(var)2-5^05/02^* third instar larvae at the promoters of *Ubx*, *hunchback* (*hb*), *nullo*, bottleneck (*bnk*), *hairy* (*h*), *twinstar* (*tsr*)*,concertina* (*cta*) and *light* (*lt*). (B) **Reduction in HP1a affects levels of PRC2 at promoters**. ChIP data for E(Z) as the representative of PRC2, in embryos from wild-type and *Su(var)2-5^05^* heterozygous parents. All promoters, except *lt*, show a reduction in signal indicating a dependence of E(Z) on HP1a for its recruitment. Non-immune (NI). All NI data is significantly below the lowest ChIP signal. Asterisks show changes which are significant relative to wild-type; * p-value <0.05, ** p-value <0.005, *** p-value <0.0005. Error bars represent +/- SEM.

**Table S1: Female viability with decreased X chromosome counting genes in presence of PcG/ trxG mutations alone and in combination with Su(var)2-5 or Su(var)3-9 mutation**

|  | **ash1^MB03235^** | **Su(z)12^4^** | **E(z)^731^** | **Suvar3-9^1^** | **Suvar2-5^05^** | **Esc^6^** | **Suvar2-5^04^** |
| --- | --- | --- | --- | --- | --- | --- | --- |
| **/ +** | ­3.3 ±1.86 | 74.6 ±2.15 | 92.5 ±1.93 | 35.4 ±1.82 | 5.0 ±2.06 | 18.7 ±2.32 | 8.74±2.30 |
| **/ ash1^MB03235^** | ND | 34.0 ±2.3 | 64.4 ±1.9 | 29.4 ±1.66 | 1.0 ±2.0 | 7.45 ±1.83 | 5.4±2.00 |
| **/ Su(z)12^4^** | 34.0 ±2.3 | ND | 91.1 ±1.79 | 89.4 ±1.87 | 54.3 ±2.13 | 54.4 ±1.82 | 86.1±2.41 |
| **/E(z) ^731^** | 64.4 ±1.9 | 91.1±1.79 | ND | 94.2 ±1.88 | 44.4 ±2.69 | 65.4 ±1.94 | 51.2±2.12 |
| **/ Suvar3-9^1^** | 29.4 ±1.66 | 89.4 ±1.87 | 94.2 ±1.88 | ND | 36.9 ±2.19 | 36.5 ±1.95 | ND |
| **/ Suvar2-5^05^** | 1.0 ±2.0 | 54.3 ±2.13 | 44.4 ±2.69 | 36.9 ±2.19 | ND | 14.6 ±2.09 | ND |
| **/ Esc^6^** | 7.45 ±1.83 | 54.4 ±1.82 | 65.4 ±1.94 | 36.5 ±1.95 | 14.6 ±2.09 | ND | 24.1±2.40 |
| **/ Suvar2-5^04^** | 5.4±2.00 | 86.1±2.41 | 51.2±2.12 | ND | ND | 24.1±2.40 | ND |
| Percent female viability ± percent Standard Error (SE). *w^1118^* alone, which serves as wild-type, was between 25-30%. ND not determined. Range of ref. male number: 231 – 698. | | | | | | | |

**Table S2: Additional PcG alleles tested for female viability effects**

|  | **Su(z)12^1^** | **Su(z)12^2^** | **E(z)^63^** |
| --- | --- | --- | --- |
| **/ +** | 39.8±1.68 | 36.3±1.98 | 85.1±1.95 |
| **/ ash1^MB03235^** | 11.8±1.84 | 10.5±1.97 | 40.1±2.02 |
| **/ esc^6^** | ND | ND | 39.1±2.13 |
| **/ Suvar3-9^1^** | 73.1±1.73 | 63.8±1.85 | 109.5±2.32 |
| **/ Suvar2-5^05^** | 26.0±1.98 | 9.9±3.0 | 23.0±2.75 |
| Percent female viability ± percent Standard Error. *w^1118^* alone, serves as wild-type was between 25-30%. ND not determined. Range of ref. male number: 221- 669. | | | |

**Table S3: Primers used in this study-related to all f**igures.

| **Primer Name** | **Primer Sequence** |
| --- | --- |
| ***in situ* hybridization primers:** |  |
| Ubx/ T7 | 5’-TAATACGACTCACTATAGGGCGAAGACTCTCTCAAACAACTC-3’ |
| Ubx R | 5’-CAAGCGTTTGAGCAGCAACCG-3’ |
| bnk/ T7 | 5’-TAATACGACTCACTATAGGGAGCACGGAGTCTTGCGTGATTC-3’ |
| bnk R | 5’-AGAGTTCCAGTGCCTCAGTACG-3’ |
|  |  |
| **promoter primers:** |  |
| +138F | 5’-[CGCTTATCCAGGGTTGCCATACCA](http://www.idtdna.com/OrderStatus/SpecSheet.aspx?OrderNum=07000000C86261D4FD2CA4E7B7D25E54713D3BD1&MfgID=08000000091F9F88C6F85E9F2EADC1859186333C&MfgLocID=1&ProdID=1213&position=7)-3’ |
| +138R | 5’-[CGGCACCCCTCTGTCGATCC](http://www.idtdna.com/OrderStatus/SpecSheet.aspx?OrderNum=07000000C86261D4FD2CA4E7B7D25E54713D3BD1&MfgID=08000000C8ED2B89157BCFD07D990FF2688F5994&MfgLocID=1&ProdID=1213&position=8)-3’ |
| *hb* F | 5’-GCCTGTCAATGCTGGCGACTTTCG-3’ |
| *hb* R | 5’-GAAAGAGCGAACGCCACGAAGGG-3’ |
| hb-P 3’ F | 5’-GGACGAAACGTAACTGCTATC-3’ |
| hb-P 3’ R | 5’-TTGACCGCGGCACGGTTACCG-3’ |
| Ubx P 5’ | 5’-CCATGATGAATTTCCCGCGGC-3’ |
| Ubx P 3’ | 5’-AGCGGTAAAGCGCTGAGGGC-3’ |
| Twin-P fwd | 5’-GTTCCAAAACAGCGAATGTGTTAAG-3’ |
| Twin-P rev | 5’-GCAAAGTATCAACATGCGTCG-3’ |
| tsr-P 3’ F | 5’-TGATACTTTGCCGGCGCCTGG-3’ |
| tsr-P 3’ R | 5’-CAATGACCGGCCGCTTTTACC-3’ |
| P-hairy 3’ F | 5’-GTAGTCCCGCTACGCTCCGC-3’ |
| P-hairy 3’ R | 5’-GAACCGCACGCTACGAAATACG-3’ |
| sgs8-P F | 5’-CTTTACCAGATGGTAACCGT-3’ |
| sgs8-P R | 5’-GAACGATGACACAGAAAATG-3’ |
| lt-P F | 5’-GCTATAACATCGAATAATAAGACCG-3’ |
| lt-P R | 5’-CTCACACCTTTAAGTGTACAGCG-3’ |
| cta-P F | 5’-GGCTGAGAGTGTCATAACCTAAT-3’ |
| cta-P R | 5’-CCCCACACTAACAGCAGATGC-3’ |
| nullo-P F | 5’-TGATCCCTATTAGGGTCCTGCCC-3’ |
| nullo-P R | 5’-GAGATGCTCAGTTTGCGGAGGACG-3’ |
| bnk-P F | 5’-CTGGACCAGGTAGTCTTTAGAAG-3’ |
| bnk-P R | 5’-AGTTGGCTGGTAAAGCGCGGC-3’ |
|  |  |
| **mRNA quantitation primers:** |  |
| tub56D 5’ | 5’-ACGAGCAGATGCTGAACATCCAGA-3’ |
| tub56D 3’ | 5’-CGGTGTACCAATGCAAGAAAGCCT-3’ |
| SxlPE mRNA F | 5’-CTGTTCGACCATGTCGTCCTAC-3’ |
| SxlPE mRNA R | 5’-CTACCACCGCTGCCCAGCGAC-3’ |
| Ubx mRNA F | 5’-GCATCCTTCGTTACGTATCTT-3’ |
| Ubx mRNA R | 5’-CAAGCGTTTGAGCAGCAACCG-3’ |
| hb mRNA F | 5’-CCATGTGCCGCAGGCCAATACC-3’ |
| hb mRNA R | 5’-CCTGGCCATGTGAACGAAGAG-3’ |
| bnk mRNA F | 5’-CCCAAGCCCAAGAGACTGTCG-3’ |
| bnk mRNA R | 5’-GGCTAACTTATAACTAAAGTAAGGC-3’ |
| hairy mRNA F | 5’-CTACCTCAGTCCAAGTACTTGG-3’ |
| hairy mRNA R | 5’-GTTCACTATATCACGTGGCC-3’ |
| Twinstar F | 5’-GTACATGCACCAGTGTCAAGGC-3’ |
| Twinstar R | 5’-CGGTGGCCCGGAGTTTCTCC-3’ |
| E(Z) mRNA F | 5’- ATCTTTCTTAAGGAGGGTGCG-3’ |
| E(Z) mRNA R | 5’-CCGTATCTGTAGTCAAAGAATAGC-3’ |
| Suvar 3-9 F | 5’CTCTTGCGATCCTAATCTCGC-3’ |
| Suvar 3-9 R | 5’-GATGTCTCAGGTGGGTAACGG-3’ |
| SetDB1 F | 5’-GCCAAACTATCACTACCAGCGC-3’ |
| SetDB1 R | 5’-TTGCCCGTTGTCTTGGCGTCC-3’ |
|  |  |
| **Other primers:** |  |
| TART F | 5’-CCGGAGTGTTTCCTGCAGAC-3’ |
| TART R | 5’-TGTTGCGTTTCGTCGTGTTC-3’ |
| 1360 F | 5’-GAGAGCGAGAGAGCGAAGAGCGC-3’ |
| 1360 R | 5’-GCGGACACAAGCACTCAACAATCATTGC-3’ |
| F-element F | 5’-ATCACGGTAGAAGAGCCGCA-3’ |
| F-element R | 5’-TGTGAAGACGGATTTTCAGCTC-3’ |
| Tre-2 F | 5’-CAAAACACGAATACAAGCCCG-3’ |
| Tre-2 R | 5’-GCTCTCGCTTTACGGCGCAG-3’ |
